# Evolutionary trends and genetic determinants of third-generation cephalosporin resistance in *Escherichia coli* from Korean livestock: a 14-year longitudinal study, 2010–2023

**DOI:** 10.3389/fmicb.2026.1816478

**Published:** 2026-05-08

**Authors:** Ji-In Kim, Ji-Hyun Choi, Md. Sekendar Ali, Bo-Youn Moon, Hee-Seung Kang, Ha-Young Kim, Yu-Jeong Hwang, Yeon-Hee Lee, Min Young Kim, Jae-Myung Kim, Tatsuya Unno, Suk-Kyung Lim

**Affiliations:** 1 Bacterial Disease Division, Animal and Plant Quarantine Agency, Ministry of Agriculture, Food and Rural Affairs, Gimcheon, Republic of Korea; 2 Department of Biological Sciences and Biotechnology, Chungbuk National University, Seowon-Gu, Cheongju, Republic of Korea

**Keywords:** *bla*_CTX − M−55_, *Escherichia coli*, livestock, multidrug resistance, third-generation cephalosporin

## Abstract

The emergence of third-generation cephalosporin-resistant *Escherichia coli* in livestock is a critical public health concern due to its potential transmission to humans via the food chain. This study investigated the 14-year longitudinal trends (2010–2023) of third-generation cephalosporin resistance, its genetic determinants, and phenotypic characteristics in *E. coli* isolated from various livestock species in South Korea. Resistance was significantly higher in chickens and pigs than in cattle, with a marked increase observed in healthy chickens over the last decade. A major shift in extended-spectrum beta-lactamase (ESBL) genotypes occurred around 2014, where *bla*_CTX − M−55_ emerged as the predominant variant (52.3%, 112/214), surpassing *bla*_CTX − M−14_ and *bla*_CTX − M−15_. The majority of isolates (88.8%, 190/214) exhibited multidrug resistance (MDR), frequently involving co-resistance to fluoroquinolones and colistin. These resistance determinants were highly transferable, primarily mediated by IncF and IncI1 plasmids, associated with mobile genetic elements such as IS*Ecp1* and IS*26*. Multi-locus sequence typing (MLST) revealed high genetic diversity, with ST752, ST457, and ST10 identified as the most prevalent lineages. Next-generation sequencing (NGS) further confirmed the presence of megaplasmids co-harboring ESBL genes, *mcr-1*, metal resistance genes, and diverse virulence factors. These findings highlight a significant evolutionary shift toward *bla*_CTX − M−55_ dominance and the rising threat of MDR *E. coli* in Korean livestock. The high transferability and virulence of these resistance determinants underscore the urgent need for continuous “One Health” surveillance and integrated antimicrobial stewardship to mitigate the risk of zoonotic transmission.

## 1 Introduction

*Escherichia coli* is a versatile facultative commensal bacterium predominantly inhabiting the gastrointestinal tracts of humans and animals. While typically harmless, it is a significant opportunistic pathogen and serves as a critical sentinel for monitoring antimicrobial resistance (AMR) trends in both human and livestock populations (ECDC and CVMP, 2017). Of particular concern is its role as a reservoir for resistance genes that can be disseminated to humans through the food chain, leading to global food poisoning outbreaks as documented in previous studies (Seo et al., 2023; Ribeiro et al., 2024; Hossain et al., 2025).

The escalating misuse and overuse of antimicrobials in the livestock industry have accelerated the emergence of resistant strains, presenting a formidable challenge to public health (Do et al., 2025). Third-generation cephalosporins (3GCs) are designated by the World Health Organization as “critically important antimicrobials” for the treatment of severe bacterial infections in both human and veterinary medicine. However, their clinical utility is increasingly compromised by the proliferation of extended-spectrum beta-lactamases (ESBLs), primarily of the CTX-M type (Hernández-Fillor et al., 2021). These enzymes confer resistance to a broad spectrum of beta-lactams, including penicillins and expanded-spectrum cephalosporins (Peirano and Pitout, 2019).

Recent global surveillance has reported a surge in ESBL-producing *E. coli* in livestock, with *bla*_CTX − M_ variants identified as the predominant genetic determinants across the USA, Europe, and Asia (Dahms et al., 2015; Tadesse et al., 2018; Widodo et al., 2020). In South Korea, while previous studies have characterized ESBL-carrying *E. coli* in food animals, most research has been constrained by small sample sizes, limited geographical coverage, or short observational periods. Furthermore, the dynamic shift in the molecular epidemiology of these genes—such as the recent emergence of *bla*_CTX − M−55_ and its association with multidrug resistance (MDR) and colistin resistance (*mcr-1*)—necessitates a more comprehensive, long-term investigation.

In this study, we provide an extensive overview of the trends in 3GC resistance in *E. coli* isolated from various livestock species in Korea over a 14-year period (2010–2023). We specifically focus on the phenotypic resistance patterns, the evolutionary transition of *bla*_CTX − M_ genotypes, and the role of mobile genetic elements in the horizontal transfer of these determinants. By employing multi-locus sequence typing (MLST) and next-generation sequencing (NGS), we aim to elucidate the genetic environment of these high-risk clones and their implications for the “One Health” framework in South Korea.

## 2 Materials and methods

### 2.1 Sample collection, isolation, and identification of *E. coli*

Sample collection, isolation, and identification of *E. coli* were conducted using the previously described protocol (Lim et al., 2015). Swab samples of healthy and diseased cattle, pigs, chickens, and duck carcasses and feces were obtained from 16 laboratories/centers that participated in the Korean Veterinary Antimicrobial Resistance Monitoring System from 2010 to 2023. The healthy carcass samples were swabbed with a sterile gauze pad (10 × 10 cm^2^) to an unconfined area of about 100 cm^2^ of the dorsal and thoracic regions of pig and cattle carcasses, whereas the entire carcasses of chickens were swabbed, followed by homogenization of the gauze in 1% buffered peptone water. The fecal samples were collected by gently rotating the sterile cotton swab in the rectum of healthy and diarrheal diseased cattle, pigs, and chickens. The isolation process of *E. coli* involves inoculating the swab sample onto MacConkey agar (Becton Dickinson, NV, USA) and incubating it overnight at 37 °C. *E. coli* was identified by matrix-assisted laser desorption ionization time-of-flight mass spectrometry (MALDI-TOF; Bruker Corporation, MA, USA). One *E. coli* isolate from each animal was selected for subsequent analysis. A total of 15,397 *E. coli* isolates were collected, comprising those from cattle (*n* = 5,252), pigs (*n* = 5,582), and chickens (*n* = 4,563). However, we do not have data on antimicrobial usage history in animals and the number of samples considered for this investigation.

### 2.2 Antimicrobial susceptibility testing

The broth microdilution method was carried out using a commercial Sensititre panel KRNVF (Thermo Fisher, Waltham, USA) to determine the antimicrobial susceptibility of the isolates against the tested antimicrobials (cefoxitin, chloramphenicol, ciprofloxacin, colistin, gentamicin, nalidixic acid, streptomycin, tetracycline, and trimethoprim/sulfamethoxazole). *E. coli* ATCC 25922 (American Type Culture Collection) served as a quality reference isolate. The obtained results were interpreted in accordance with the [Clinical and Laboratory Standards Institute (CLSI), 2023]. Moreover, antimicrobial susceptibility to third-generation cephalosporins was determined using standard methods, and resistance trends were analyzed across different livestock species and over time. In addition, the antimicrobial resistance profiles of *bla*_CTX − M−55_-carrying *E. coli* were compared to those carrying other *bla*_CTX − M_ types against a panel of various antimicrobial agents, including chloramphenicol and colistin.

### 2.3 Identification of ESBL genes

Polymerase chain reaction (PCR) was performed on genomic DNA extracted from a total of 214 *E. coli* strains that were resistant to third-generation cephalosporin using specific primers for ESBLs to identify the particular *bla*_CTX − M_ gene types present in the isolates. The sequencing of PCR products was carried out using the ABI3730XL DNA sequencing analyzer (SolGent, Daejeon, South Korea), and the sequences were verified by comparing them with the known sequences in the GenBank database by the Basic Local Alignment Search Tools (BLAST) on the National Center for Biotechnology Information website (https://www.ncbi.nlm.nih.gov/BLAST). The primers and PCR conditions are detailed in Supplementary material (Supplementary Table 1).

### 2.4 Conjugation assay and replicon typing

The conjugation assay was conducted to evaluate the transferability of β-lactamase genes using solid mating experiments with sodium azide-resistant *E. coli* J53 as the recipient strain (Ji-In et al., 2025). Briefly, the freshly cultivated bacteria were mated at a 1:4 ratio of donor to recipient, followed by culture on tryptic soy agar plates overnight and subsequently selected on MacConkey agar plates supplemented with sodium azide (150 μg/mL) and cefotaxime (2 μg/mL). All selected transconjugants were evaluated for the presence of β-lactamase genes and antimicrobial susceptibility. Additionally, the co-transfer of resistance to other antimicrobial classes in transconjugants was investigated using disc diffusion based on CLSI guidelines [Clinical and Laboratory Standards Institute (CLSI), 2023]. The replicon types of plasmids carrying *bla*_CTX − M_ genes in transconjugants were identified by multiplex PCR using specific primers and PCR conditions (Supplementary Table 1).

### 2.5 Molecular characterization of *bla*_*CTX*−*M*_ gene environment

Multiplex PCR and Sanger sequencing analysis were performed to determine the genetic environment of the ESBL genes in extracted DNA, following the previously described methodology. We used specific primers targeting the upstream and downstream environments of the β-lactamase genes (IS*Ecp1, Orf477*, IS*26*, and IS*903*), in addition to ESBL gene primers (Supplementary Table 1).

### 2.6 Multi-locus sequence typing (MLST)

MLST was conducted on a subset of third-generation cephalosporin-resistant *E. coli* isolates (*n* = 214) to characterize their genetic diversity using the method delineated by (Ji-In et al. 2025). Seven housekeeping genes (*adk, fumC, gyrB, icd, mdh, purA*, and *recA*) were amplified and sequenced, and allelic profiles of *E. coli* isolates were used to assign them to STs based on a web-based MLST database (https://pubmlst.org/database/).

### 2.6 Next-generation sequencing analysis

We performed next-generation sequencing (NGS) on *E. coli* isolates using the Illumina MiSeq (Illumina Inc., CA, USA) and Oxford Nanopore (Nanopore Technologies Ltd., Oxford, UK) systems. Libraries were created for short-read sequencing using the Illumina DNA Prep (M) Tagmentation kit (Illumina Inc., CA, USA), and the Illumina MiSeq platform was used to sequence the library by outsourcing to Macrogen (Macrogen Inc., Seoul, South Korea). In-house preparation of long-read libraries was conducted with the Ligation Sequencing Kit V14 (SQK-LSK114) (Nanopore Technologies Ltd., Oxford, UK). Sequencing was conducted in-house utilizing a MinION instrument, followed by basecalling with Dorado (version 1.3.1) to produce the complete genome sequence. We carried out quality control of the Illumina and Nanopore sequencing data and successively performed hybrid assembly utilizing Unicycler (version 0.4.8) (Wick et al., 2017). Genome annotation was accomplished via Prokka v1.14.6 (Seemann, 2014). ResFinder (version 2.1) was used to identify antimicrobial resistance genes from the Comprehensive Antimicrobial Resistance Database, and the Virulence Factor Database (version 5.0) was used to evaluate virulence factors. Additionally, Proksee (https://proksee.ca/) was used to analyze the features of circular genome maps, mobile genetic elements, and visualization.

### 2.7 Statistical analysis

The Rex Software (version 3.0.3; RexSoft, Inc., Seoul, South Korea), including the chi-square test, was used to analyze the obtained data. A *p*-value ≤ 0.05 was deemed statistically significant.

## 3 Results

### 3.1 Prevalence of third-generation cephalosporin resistance in *E. coli*

Overall, third-generation cephalosporin resistance in *E. coli* was found to be low in cattle (*n* = 55) but considerably higher in pigs (*n* = 331) and chickens (*n* = 486), totaling 872 during the study period (Table 1). Resistance was notably elevated in *E. coli* isolated from diseased livestock compared to healthy animals. While resistance in healthy pigs and chickens remained below 5% in the early 2010s, it significantly increased to over 10% in healthy chickens in recent years.

**Table 1:** Prevalence of third-generation cephalosporin-resistant *Escherichia coli* isolated from livestock during 2010–2023 in South Korea.

Year	% (No. of resistant isolates/No. of tested *E. coli* isolates)															
	**Cattle**				**Pigs**				**Chickens**				**Total**			**Total**
	**Healthy**	**Carcass**	**Diseased**	**Subtotal**	**Healthy**	**Carcass**	**Diseased**	**Subtotal**	**Healthy**	**Carcass**	**Diseased**	**Subtotal**	**Healthy**	**Carcass**	**Diseased**	
2010	0 (0/231)	0 (0/136)	4 (1/25)	0.3 (1/392)	0.5 (1/221)	0 (0/124)	16.1 (9/56)	2.5 (10/401)	3.2 (5/155)	3.7 (4/107)	20.0 (10/50)	6.1 (19/312)	1 (6/607)	1.1 (4/367)	15.3 (20/131)	2.7 (30/1,105)
2011	0 (0/347)	0 (0/190)	0 (0/14)	0 (0/551)	0.4 (1/231)	0 (0/153)	2.8 (1/36)	0.5 (2/420)	5.0 (7/141)	4.1 (5/121)	9.3 (4/43)	5.2 (16/305)	1.1 (8/719)	1.1 (5/464)	5.4 (5/93)	1.4 (18/1,276)
2012	0.4 (1/282)	0.9 (1/111)	0 (0/28)	0.5 (2/421)	0 (0/277)	0.7 (1/134)	5.1 (2/39)	0.7 (3/450)	4.0 (8/200)	6.7 (11/163)	25.0 (2/8)	5.7 (21/371)	1.2 (9/759)	3.2 (13/408)	5.3 (4/75)	2.1 (26/1,242)
2013	0 (0/209)	0 (0/96)	0 (0/5)	0 (0/310)	0.5 (1/199)	2.1 (2/96)	17.9 (5/28)	2.5 (8/323)	8.6 (16/187)	7.5 (8/107)	9.1 (3/33)	8.3 (27/327)	2.9 (17/595)	3.3 (10/299)	12.1 (8/66)	3.6 (35/960)
2014	1.7 (5/299)	0 (0/159)	0 (0/8)	1.1 (5/466)	2.0 (6/294)	1.3 (2/160)	18.4 (7/38)	3.0 (15/492)	6.8 (13/192)	15.0 (21/140)	7.7 (3/39)	10.0 (37/371)	3.1 (24/785)	5.0 (23/459)	11.8 (10/85)	4.3 (57/1,329)
2015	1.9 (4/206)	0 (0/125)	5.3 (1/19)	1.4 (5/350)	10.6 (23/218)	1.8 (2/111)	5.8 (9/154)	7.0 (34/483)	9.5 (18/189)	13.4 (17/127)	25 (5/20)	11.9 (40/336)	7.3 (45/613)	5.2 (19/363)	7.8 (15/193)	6.8 (79/1,169)
2016	0.2 (1/401)	0.9 (2/218)	3.8 (1/26)	0.6 (4/645)	4.0 (14/347)	2.1 (5/238)	10.9 (5/46)	3.8 (24/631)	10.6 (32/303)	10.7 (19/177)	23.7 (9/38)	11.6 (60/518)	4.5 (47/1051)	4.1 (26/633)	13.6 (15/110)	4.9 (88/1,794)
2017	2.3 (6/263)	3.8 (5/131)	4.9 (2/41)	3.0 (13/435)	5.3 (14/262)	6.9 (10/145)	7.0 (4/58)	6.0 (28/465)	13.1 (18/137)	12.3 (19/154)	25.9 (7/27)	13.8 (44/318)	5.7 (38/662)	7.9 (34/430)	10.3 (13/126)	7.0 (85/1,218)
2018	0 (0/178)	0 (0/74)	0 (0/64)	0 (0/316)	4.8 (9/189)	4.3 (3/70)	25.5 (41/161)	12.6 (53/420)	11.7 (19/163)	9.9 (8/81)	11.1 (7/63)	11.1 (34/307)	5.3 (28/530)	4.9 (11/225)	16.7 (48/288)	8.3 (87/1,043)
2019	1.3 (2/152)	1.6 (1/62)	3 (1/33)	1.6 (4/247)	10.8 (15/139)	10.5 (6/57)	23.7 (18/76)	14.3 (39/272)	12.6 (18/143)	17.7 (14/79)	12.5 (5/40)	14.1 (37/262)	8.1 (35/434)	10.6 (21/198)	16.1 (24/149)	10.2 (80/781)
2020	0 (0/165)	0 (0/71)	17.6 (3/17)	1.2 (3/253)	5.5 (9/165)	11.0 (9/82)	14.8 (13/88)	9.3 (31/335)	11.8 (18/152)	11.3 (9/80)	15.9 (11/69)	12.6 (38/301)	5.6 (27/482)	7.7 (18/233)	15.5 (27/174)	8.1 (72/889)
2021	1.2 (2/170)	2.8 (2/71)	0 (0/42)	1.4 (4/283)	10.2 (17/167)	12.9 (8/62)	13.1 (8/61)	11.4 (33/290)	10.1 (15/149)	13.3 (11/83)	13.2 (9/68)	11.7 (35/300)	7.0 (34/486)	9.7 (21/216)	9.9 (17/171)	8.2 (72/873)
2022	1.3 (2/156)	5.3 (4/76)	6.5 (3/46)	3.2 (9/278)	7.1 (11/155)	7.1 (5/70)	6.6 (4/61)	7.0 (20/286)	13.1 (20/154)	17.2 (16/93)	27.8 (10/36)	16.3 (46/283)	7.1 (33/465)	10.5 (25/239)	11.9 (17/143)	8.9 (75/847)
2023	0.6 (1/170)	1.6 (1/62)	4.1 (3/73)	1.6 (5/305)	8.5 (14/165)	4.3 (3/70)	17.7 (14/79)	9.9 (31/314)	9.6 (13/135)	19.5 (16/82)	8.6 (3/35)	12.7 (32/252)	6.0 (28/470)	9.3 (20/214)	10.7 (20/187)	7.8 (68/871)
Total	0.7 (24/3,229)	1 (16/1,582)	3.4 (15/441)	1.0 (55/5,252)	4.5 (135/3,029)	3.6 (56/1,572)	14.3 (140/981)	5.9 (331/5,582)	9.2 (20/2,400)	11.2 (178/1,594)	15.5 (88/569)	10.7 (486/4,563)	4.4 (379/8,658)	5.3 (250/4,748)	12.2 (243/1,991)	5.7 (872/15,397)

### 3.2 Distribution of *bla*_CTX − M_ genes

A total of nine distinct *bla*_CTX − M_ gene variants were identified across all livestock species (Table 2). The CTX-M-1 group was predominant, accounting for six of the identified genes and approximately 80% (178/214) of all *bla*_CTX − M_ genes detected. The CTX-M-9 group comprised the remaining two identified genes. *bla*_CTX − M−55_ was the most prevalent gene, accounting for approximately 53% (112/214) of all detected *bla*_CTX − M_ genes. This represents a significant shift from the distribution observed before 2014, when *bla*_CTX − M−14_ and *bla*_CTX − M−15_ were the primary variants. Since its emergence in 2014, *bla*_CTX − M−55_ has consistently accounted for around 50% of the total *bla*_CTX − M_ genes each year, particularly in pigs and chickens. *bla*_CTX − M−14_ and *bla*_CTX − M−15_ collectively comprised about 30% (63/112) of the total *bla*_CTX − M_ genes. Together, *bla*_CTX − M−55_, *bla*_CTX − M−14_, and *bla*_CTX − M−15_ accounted for over 80% (175/214) of all *bla*_CTX − M_ genes observed in the livestock isolates.

**Table 2:** Distribution of ESBL genes in *Escherichia coli* isolated from livestock during 2010–2023 in South Korea.

Year	CTX-M-1 group								CTXM-9 group		
	*bla*_CTX − M−1_	*bla*_CTX − M−3_	*bla*_CTX − M−8_	*bla*_CTX − M−15_	*bla*_CTX − M−55_	*bla*_CTX − M−65_	*bla*_CTX − M−101_	**Subtotal (%)**	*bla*_CTX − M−14_	*bla*_CTX − M−27_	**Subtotal (%)**
2011	–	–	–	4	–	–	–	4 (80.0)	1	–	1 (20.0)
2012	1	–	–	3	–	–	–	4 (80.0)	1	–	1 (20.0)
2013	–	–	–	2	–	1	–	3 (50.0)	3	–	3 (50.0)
2014	1	–	–	1	7	1	–	10 (90.9)	1	–	1 (9.1)
2015	3	–	–	8	17	4	–	32 (88.9)	3	1	4 (11.1)
2016	4	2	–	2	15	–	–	23 (92.0)	2	–	2 (8.0)
2017	3	1	–	1	5	1	–	11 (78.6)	3	–	3 (21.4)
2018	–	–	–	-	10	2	–	12 (66.7)	4	2	6 (33.3)
2019	1	–	1	2	15	1	–	20 (80.0)	5	–	5 (20.0)
2020	2	–	–	1	10	1	–	14 (87.5)	2	–	2 (12.5)
2021	–	–	–	3	14	–	–	17 (89.5)	2	–	2 (10.5)
2022	1	1	–	1	12	1	–	16 (100.0)	–	–	0 (0)
2023	–	–	–	4	7	–	1	12 (66.7)	4	2	6 (33.3)
Total	16 (7.5)	4 (1.9)	1 (0.5)	32 (15.0)	112 (52.3)	12 (5.6)	1 (0.5)	178 (83.2)	31 (14.5)	5 (2.3)	36 (16.8)

The overall distribution of CTX-M types showed little significant variation across livestock species (Supplementary Tables 2–4). While *bla*_CTX − M−55_ was the most abundant in all species, *bla*_CTX − M−14_ and *bla*_CTX − M−15_ were also detected in bovine, porcine, and avian isolates. Notably, *bla*_CTX − M−1_ was found at very low levels in bovine and porcine isolates, but accounted for approximately 13% (15/112) in chickens.

### 3.3 Antimicrobial resistance in *bla*_*CTX*−*M*_-carrying *E. coli*

Overall, CTX-M-producing *E. coli* isolates exhibited high levels of co-resistance to non-β-lactam antimicrobials (Table 3). Resistance rates were particularly high (exceeding 60%) for chloramphenicol, ciprofloxacin, nalidixic acid, streptomycin, and tetracycline. Variations in antimicrobial resistance were observed depending on the specific ESBL type. Isolates carrying *bla*_CTX − M−55_ showed significantly higher resistance to cefoxitin compared to other ESBL types. *bla*_CTX − M−1_ and *bla*_CTX − M−14_, which were primarily found in chickens, exhibited high resistance (over 70%) to (fluoro)quinolone antimicrobial. Of note, colistin resistance was identified in 1.8% (2/112) of *bla*_CTX − M−55_ and 2.9% (3/102) of the other *bla*_CTX − M_-carrying *E. coli* isolates. We found that 88.8% (190/214) of the isolates showed MDR. Among them, most *bla*_CTX − M−55_-carrying (89.3%, 100/112) and other *bla*_CTX − M_-carrying (88.2%, 90/102) isolates demonstrated MDR (Supplementary Table 5). In addition, different combination patterns were detected in *bla*_CTX − M_-carrying *E. coli* isolates. Moreover, these patterns frequently contain five or more antimicrobial resistance, including chloramphenicol, ciprofloxacin, nalidixic acid, streptomycin, and tetracycline.

**Table 3:** Antimicrobial resistance of ESBL-harboring *Escherichia coli* isolated from livestock during 2010–2023 in South Korea.

Antimicrobials	Antimicrobial resistance % (No. of isolates)								*p-*value
	*bla*_CTX − M−55_ **(*****n*** = **112)**	**Other** *bla*_CTX − M_						**Total (*****n*** = **214)**	
		*bla*_CTX − M−1_ **(*****n*** = **16)**	*bla*_CTX − M−14_ **(*****n*** = **31)**	*bla*_CTX − M−15_ **(*****n*** = **32)**	*bla*_CTX − M−65_ **(*****n*** = **12)**	**Other** *bla*_CTX − M_ **(*****n*** = **11)**	**Subtotal (*****n*** = **102)**		
Cefoxitin	12.5 (14)	0 (0)	0 (0)	12.5 (4)	0 (0)	0 (0)	3.9 (4)	8.4 (18)	0.02
Chloramphenicol	76.8 (86)	50.0 (8)	64.5 (20)	50.0 (16)^*^	91.7 (11)	72.7 (8)	61.8 (63)	69.6 (149)	0.01
Ciprofloxacin	60.7 (68)	81.3 (13)	61.3 (19)	46.9 (15)	66.7 (8)	36.4 (4)	57.8 (59)	59.3 (127)	0.66
Colistin	1.8 (2)	6.3 (1)	0 (0)	3.1 (1)	0 (0)	9.1 (1)	2.9 (3)	2.3 (5)	0.20
Gentamicin	25.0 (28)	18.8 (3)	61.3 (19)^*^	43.8 (14)	50.0 (6)	18.2 (2)	43.1 (44)	33.6 (72)	< 0.01
Nalidixic acid	75.9 (85)	93.8 (15)	77.4 (24)	71.9 (23)	91.7 (11)	54.5 (6)	77.5 (79)	76.6 (164)	0.78
Streptomycin	65.2 (73)	68.8 (11)	64.5 (20)	81.3 (26)	75.0 (9)	72.7 (8)	72.5 (74)	68.7 (147)	0.24
Tetracycline	67.9 (76)	93.8 (15)^*^	58.1 (18)	81.3 (26)	66.7 (8)	72.7 (8)	73.5 (75)	70.6 (151)	0.36
Trimethoprim/sulfamethoxazole	42.0 (47)	31.3 (5)	51.6 (16)	53.1 (17)	75.0 (9)	63.6 (7)	52.9 (54)	47.2 (101)	0.10
MDR	89.3 (100)	93.8 (15)	87.1 (27)	84.4 (27)	100.0 (12)	81.8 (9)	88.2 (90)	88.8 (190)	0.80

^*^Significant difference compared to total.

MDR, multidrug resistance.

### 3.4 Third-generation cephalosporin resistance transfer and transconjugant characteristics

The transferability varied significantly among CTX-M types (Table 4). Notably, *bla*_CTX − M−1_ exhibited a high transfer rate of 68.8% (11/16). The three most common types (*bla*_CTX − M−55_, *bla*_CTX − M−14_, and *bla*_CTX − M−15_) showed transfer rates ranging from 9.4% to 31.3%. Replicon typing of transconjugants revealed diverse plasmid types, with IncF (24.8%, 53/214), IncFIB (13.6%, 29/214), IncI1 (12.1%, 26/214), and IncFIC (7.5%, 16/214) replicons frequently observed, although variations existed among different *bla*_CTX − M_ types. Among them, the plasmid types IncFIB (65.7%, 23/35), IncFIC (57.1%, 20/35), and IncF (91.4%, 32/35) are mainly detected in *bla*_CTX − M−55_ transconjugants. Moreover, IncI1 was predominantly identified in *bla*_CTX − M−1_ (100%, 11/11), *bla*_CTX − M−14_ (33%, 3/9), and *bla*_CTX − M−55_ (28.6%, 10/35) transconjugants_._ Investigation of transconjugant antimicrobial resistance profiles revealed that approximately 69.4% of the transferred resistance included not only β-lactams but also co-resistance to at least one other antimicrobial class. Transconjugants frequently displayed co-resistance to commonly used veterinary antimicrobials, such as tetracycline, phenicol (chloramphenicol), and aminoglycosides.

**Table 4:** Characterization of β-lactamase genes in *Escherichia coli* isolated from livestock during 2010–2023 in South Korea.

*bla*_CTX − M_ types	No. of isolates (%)			Transferability % (No. of transferred/No. of tested isolates)	Replicon type	Transferred resistance (non-bate lactam)
	Cattle	Pigs	Chickens			
*bla*_CTX − M−1_ (*n* = 16)	0 (0)	1 (6.3)	15 (93.8)	68.8 (11/16)	I1, F (*n* = 5)	TET (*n* = 3), CHL-TET (*n* = 1)
					I1 (*n* = 2)	–
					I1, FIB, F (*n* = 2)	TET (*n* = 1)
					I1, FIB, FIC, F (*n* = 1)	TET(*n* = 1)
					I1, F, B/O (*n* = 1)	CHL-TET (*n* = 1)
*bla*_CTX − M−3_ (*n* = 4)	0 (0)	3 (75.0)	1 (25.0)	25.0 (1/4)	I1, F (*n* = 1)	CHL-TET-STR (*n* = 1)
*bla*_CTX − M−8_ (*n* = 1)	0 (0)	0 (0)	1 (100)	0 (0/1)	–	–
*bla*_CTX − M−14_ (*n* = 31)	2 (6.5)	9 (29.0)	20 (64.5)	29.0 (9/31)	F (*n* = 3)	GEN (*n* = 1), CHL-GEN-SXT (*n* = 1)
					B/O (*n* = 1)	-
					I1 (*n* = 1)	CHL-GEN (*n* = 1)
					HI2, F (*n* = 1)	CHL-SXT (*n* = 1)
					HI2, I1, FIB, FIC, F (*n* = 1)	CHL-SXT (*n* = 1)
					I1, F (*n* = 1)	CHL-SXT (*n* = 1)
					K2 (*n* = 1)	GEN (*n* = 1)
*bla*_CTX − M−15_ (*n* = 32)	3 (9.4)	15 (46.9)	14 (43.8)	9.4 (3/32)	I1, F (*n* = 1)	TET-GEN-STR-SXT (*n* = 1)
					NT (*n* = 2)	CHL-STR (*n* = 1)
*bla*_CTX − M−27_ (*n* = 5)	0 (0)	2 (40.0)	3 (60.0)	40.0 (2/5)	F (*n* = 1)	–
					FIA, FIB, FIC, F (*n* = 1)	–
*bla*_CTX − M−55_ (*n* = 112)	6 (5.4)	51 (45.5)	55 (49.1)	31.3 (35/112)	FIB, FIC, F (*n* = 11)	CHL (*n* = 2), CHL-TET (*n* =2), CHL-GEN (*n* = 1), CHL-SXT (*n* = 1), CHL-STR-SXT (*n* = 1), CHL-TET-SXT (*n* = 1), TET-GEN-STR (*n* = 1), CHL-TET-GEN-STR-SXT (*n* = 1)
					I1, FIB, FIC, F (*n* = 8)	TET-STR (*n* = 1), TET-GEN (*n* = 1), TET-STR-SXT (*n* = 2), CHL-TET-STR (*n* = 1), CHL-TET-STR-SXT (*n* = 1), CHL-TET-GEN-STR (*n* = 1)
					F (*n* = 5)	SXT (*n* = 1), CHL-TET-STR (*n* = 1)
					FIB, F (*n* = 3)	CHL-TET (*n* = 1), GEN-STR (*n* = 1), CHL-TET-STR-SXT (*n* = 1)
					*n*, F (*n* = 2)	–
					I1 (*n* = 1)	CHL-TET-STR-SXT (*n* = 1)
					*n*, FIB, FIC, F (*n* = 1)	–
					I1, F (*n* = 1)	–
					F, B/O (*n* = 1)	CHL-TET-STR (*n* = 1)
					NT (*n* = 2)	–
*bla*_CTX − M−65_ (*n* = 12)	1 (8.3)	3 (25.0)	8 (66.7)	16.7 (2/12)	F (*n* = 1)	SXT (*n* = 1)
					FIB, FIC, F (*n* = 1)	TET-STR-SXT(*n* = 1)
*bla*_CTX − M−101_ (*n* = 1)	0 (0)	1 (100.0)	0 (0)	100.0 (1/1)	I1 (*n* = 1)	–
Total (*n* = 214)	12 (5.6)	85 (39.7)	117 (54.7)	29.9 (64/214)		

CHL, chloramphenicol; GEN, gentamicin; STR, streptomycin; SXT, trimethoprim/sulfamethoxazole; TET, tetracycline.

**Figure 1:**
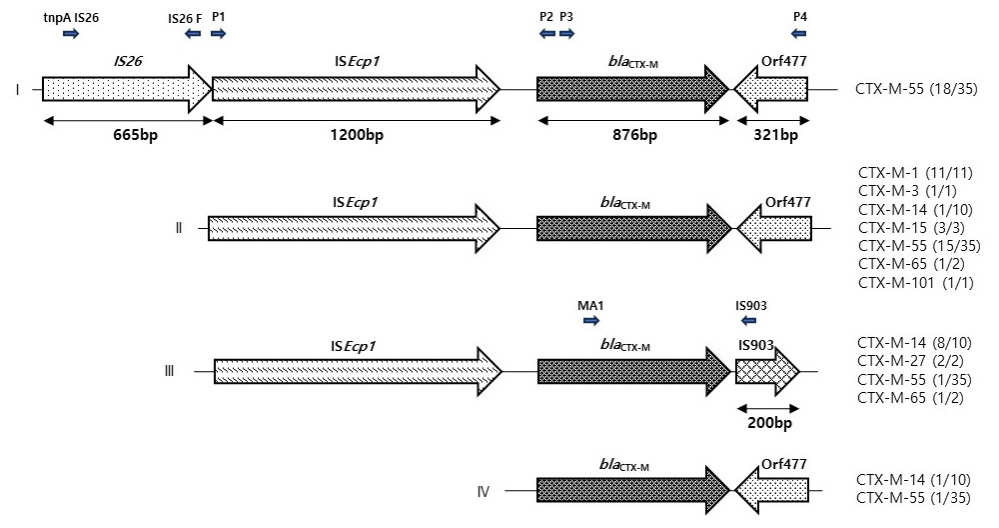
Determination of the transposable elements in the *bla*_CTX − M_ gene environment of ESBL-harboring *Escherichia coli*.

We detected genetic environments in 64 transconjugants, and subsequently clustering yielded five types of genetic structures (I–V) (Figure 1). These genetic variants were different based on the *bla*_CTX − M_ types. Type II was the most prevalent, comprising IS*Ecp1* upstream and *Orf477* downstream. This type was mostly identified in *bla*_CTX − M−1_ (100%, 11/11), *bla*_CTX − M−3_ (100%, 1/1), *bla*_CTX − M−15_ (100%, 3/3), *bla*_CTX − M−101_ (100%, 1/1), *bla*_CTX − M−55_ (42.9%, 15/35), and *bla*_CTX − M−65_ (50%, 1/2). Another commonly found type was type I, encompassing IS*26* and IS*Ecp1* upstream and *Orf477* downstream, which was identified only in *bla*_CTX − M−55_ (51.4%, 18/35). We also found type III, which includes IS*Ecp1* upstream and IS*903* downstream of *bla*_CTX − M−27_ (100%, 2/2), *bla*_CTX − M−14_ (80%, 8/10), *bla*_CTX − M−65_ (1/2, 50%), and *bla*_CTX − M−55_ (2.9%, 1/35).

### 3.5 MLST of third-generation cephalosporin-resistant *E. coli*

**Figure 2:**
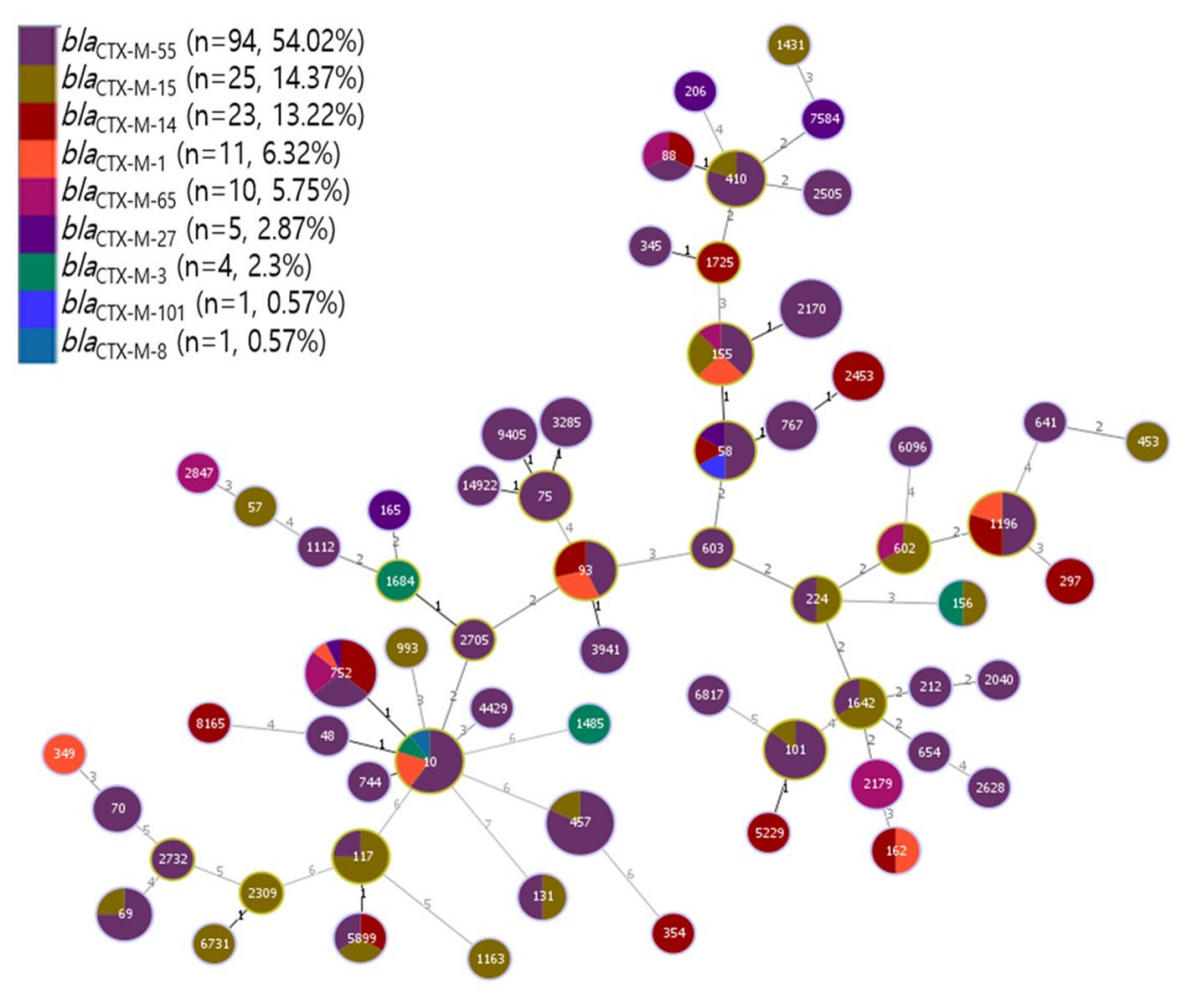
Multi-locus sequence typing (MLST) profiles of ESBL-harboring *Escherichia coli* with a phylogenetic tree demonstrating genetic relationship among STs.

MLST analysis of 214 third-generation cephalosporin-resistant *E. coli* strains showed considerable genetic diversity (Figure 2 and Supplementary Table 6). A total of 174 strains (81.3%) belonged to 64 distinct STs, while 40 strains (18.7%) could not be assigned to a known ST. The most frequently identified STs were ST752 (6.5%, 14/214), followed by ST457 (5.1%, 11/214), and ST10 and ST1196 (both 4.7%, 10/214). Furthermore, 34 STs were detected in only a single isolate. Significant variations were observed in the distribution of STs among different CTX-M types. Specifically, *bla*_CTX − M−55_ was predominantly associated with ST457, ST2170, and ST10, while *bla*_CTX − M−14_ and *bla*_CTX − M−65_ were primarily found in ST752.

Phylogenetic analysis revealed a substantial number of STs demonstrating close genetic relationships with ST10 (Figure 2). Notably, ST10 was detected in isolates harboring *bla*_CTX − M−55_, *bla*_CTX − M−1_, *bla*_CTX − M−3_, and *bla*_CTX − M−101_. Furthermore, ST410 was identified in isolates carrying the *bla*_CTX − M−55_ and *bla*_CTX − M−15_ genes.

### 3.6 Next-generation sequencing

**Figure 3:**
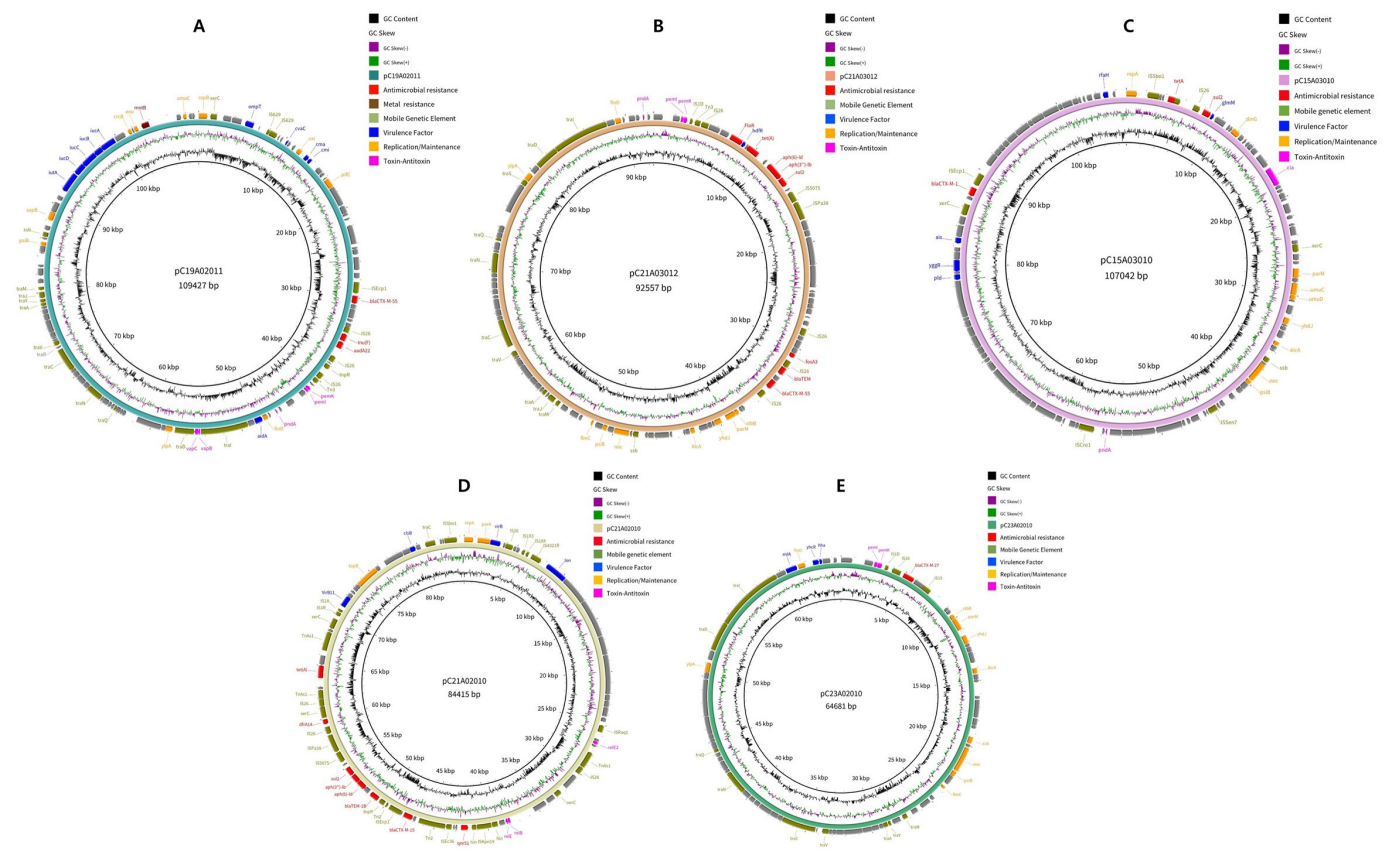
Circular plasmid maps of pC19A02021 **(A)**, pC21A03012 **(B)**, pC15A3010 **(C)**, pC21A02010 **(D)**, and pC23A02010 **(E)** carrying *bla*_CTX − M−55_, *bla*_CTX − M−55_, *bla*_CTX − M−1_, *bla*_CTX − M−15_, *bla*_CTX − M−27_, respectively in *Escherichia coli* isolates obtained from livestock.

**Figure 4:**
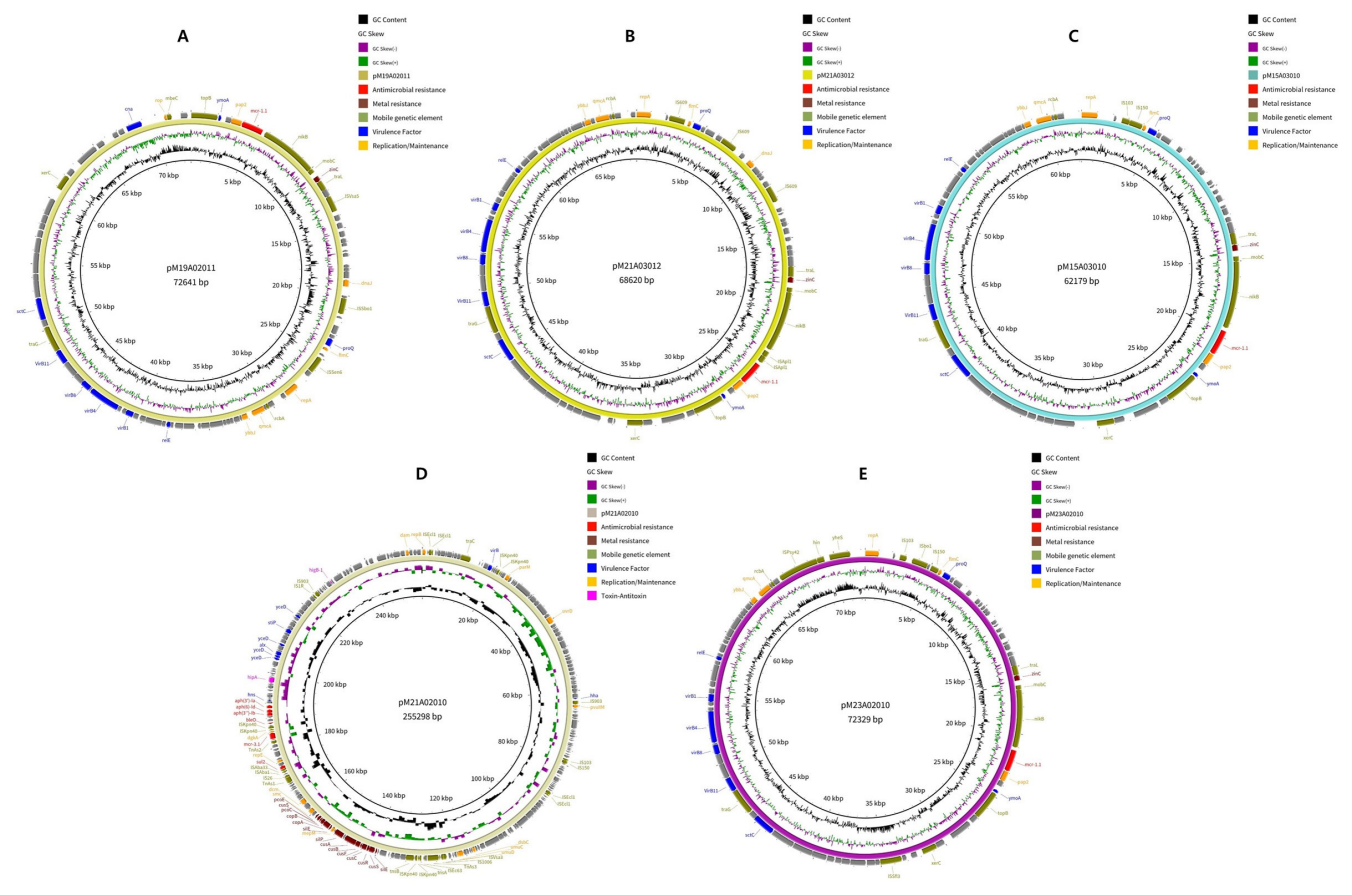
Circular plasmid maps of pM19A02021 **(A)**, pM21A03012 **(B)**, pM15A3010 **(C)**, pM21A02010 **(D)**, and pMC23A02010 **(E)** carrying *mcr-1.1, mcr-1.1, mcr-1, mcr-3.1*, and *mcr-1*, respectively in *Escherichia coli* isolates obtained from livestock.

Five *E. coli* strains were subjected to NGS; all of those strains were shown to carry both *bla*_CTX − M_ and *mcr* genes (Figures 3, 4 and Supplementary Table 7). The genome sequencing data were deposited in the GenBank database (https://www.ncbi.nlm.nih.gov/) under the NCBI accession code for pC19A02021 (SAMN56646416), pC21A03012 (SAMN56646523), pC15A3010 (SAMN56646524), pC21A02010 (SAMN56646525), and pC23A02010 (SAMN56646527). Our results showed that all five isolates from pigs and chickens contain megaplasmids approximately 500 kbp in size. A wide range of antimicrobial resistance genes, including those for beta-lactams (*bla*_CTX − M−55_, *bla*_TEM − 1_, *bla*_CTX − M−15_, and *bla*_CTX − M−27_), aminoglycosides (*aph3”)-Ib, aph(6)-Id*, and *aadA22*), sulfonamides (*sul2*), phenicols (*floR*), tetracyclines (*tet(A)*), and colistin (*mcr-1*) were present in the plasmids. In addition, the plasmids harbor various metal resistance genes, including *copA, cueR, zinC*, and *arsB*. Furthermore, a point mutation in the chromosomal DNA results in amino acid substitutions at S83L and D87Y in *gyrA*, S80I in *parC*, and S458A in *parE*, all of which are associated with quinolone resistance. A diverse set of mobile genetic elements, including IS*Ecp1*, IS*Ec12*, and IS*26*, was also found in the plasmids.

Analysis of virulence factors using the VFDB database revealed the presence of numerous virulence factors. The identified virulence genes included those associated with adhesion: *papA, papB, papC, papD, papE, papG, papH*, and *papK*; adhesion and colonization: *lpfA, lpfB, lpfC, lpfD*, and *lpfE*; fimbriae: *fimA, fimC, fimD, fimF, fimH*, and *fimI*; and stress resistance: *sodCI*. In addition to these, plasmid maintenance and replication genes (*dnaA, recB*, and *pap2*) and toxin-antitoxin genes (*mazE, relB, vapC*, and *pndA*) were present.

## 4 Discussion

In this study, we found that the predominance of *bla*_CTX − M−55_, along with the significant contributions of *bla*_CTX − M−14_ and *bla*_CTX − M−15_, confirms the prevalent role of these specific ESBL genes in mediating third-generation cephalosporin resistance in Korean livestock. Moreover, the resistance patterns observed between *bla*_CTX − M−55_ and other *bla*_CTX − M_ types for chloramphenicol and colistin are important for guiding treatment strategies and understanding the co-selection pressures.

The emergence of third-generation cephalosporin resistance in Enterobacterales, particularly *E. coli*, poses a significant threat to both humans and animals. We found that the resistance to the third-generation cephalosporins was higher in chickens and pigs than in cattle. Moreover, this resistance steadily increased throughout the investigation period. In accordance with this study, a significant prevalence of extended-spectrum cephalosporin resistance was observed in *E. coli* isolates obtained from chickens and pigs in Korea (Song et al., 2020), China (Wang et al., 2022), and Denmark (Che et al., 2023). Furthermore, the resistance was notably higher in *E. coli* isolates from diseased livestock compared to healthy animals. This might be because third-generation cephalosporins were approved for veterinary use to treat various Gram-negative bacterial infections in livestock. In Korea, these antimicrobials are commonly used in food animals, triggering the development of resistance in *E. coli* [Animal and Plant Qurantine Agency (APQA), 2023]. Cephalosporin resistance often develops when bacteria evolve in response to the frequent use of this antimicrobial.

The third-generation cephalosporin often triggers the emergence of ESBL β-lactamase genes in *Enterobacteriaceae*. Recently, the occurrence of ESBL β-lactamase-carrying *E. coli* has been increasing in both humans and livestock. In this investigation, different ESBL genes, mainly *bla*_CTX − M_ types, including *bla*_CTX − M−55_, *bla*_CTX − M−15_, *bla*_CTX − M−14_, and *bla*_CTX − M−1_, were predominantly detected. *bla*_CTX − M−1_, found in 7.5% of the *E. coli* isolates, has previously been documented in different food animals in Korea (Song et al., 2020). Moreover, *bla*_CTX − M−1_ is the leading CTX-M type among *Enterobacteriaceae* isolates from chickens and pigs in Asia (Wibisono et al., 2020) and Europe (Silva et al., 2019). The *bla*_CTX − M−14_ and *bla*_CTX − M−15_ are identified as the major *bla*_CTX − M_ genes in livestock, including cattle, chickens, and pigs, in Switzerland (Aebi et al., 2023) and Thailand (Lay et al., 2021). In South Korea, these genes were detected as the predominant CTX-M type in livestock (Hong et al., 2019; Song et al., 2020). The *bla*_CTX − M−55_ gene was also frequently identified in *E. coli* from food animals in China (Zheng et al., 2012) and Vietnam (Bui et al., 2018). Similarly, this gene is more often detected in pigs and chickens than other *bla*_CTX − M_ genes in South Korean livestock (Song et al., 2020).

We found that different non-β-lactam antimicrobial resistance patterns were transmitted along with *bla*_CTX − M_-carrying *E. coli* isolates. Among them, resistance patterns comprising aminoglycosides, tetracycline, and phenicol were commonly linked to *bla*_CTX − M−1_, *bla*_CTX − M−14_, and *bla*_CTX − M−55_. Previous studies have shown that *E. coli* from livestock possess resistance to these antimicrobials, often transmitted with the *bla*_CTX − M_ gene (Song et al., 2020; Seo et al., 2023). Specifically, *E. coli* isolates from food animals carrying the *bla*_CTX − M−55_ gene frequently transfer resistance to chloramphenicol, tetracycline, and streptomycin (Pazra and Iryawati, 2025). Furthermore, increased resistance in *E. coli* to these antimicrobials was found to be associated with the horizontal transmission of the *bla*_CTX − M_ gene, along with various other resistance genes, including *floR, tet*A, and *aac(3)-IV* (Murray et al., 2021). Hence, the co-resistance to commonly used veterinary antibiotics, including tetracyclines, phenicols, and aminoglycosides, is particularly concerning, as it further limits treatment options and contributes to the overall burden of multidrug resistance in livestock.

Differences in antimicrobial resistance were found based on the different ESBL-type genes. Isolates carrying *bla*_CTX − M−55_ showed significantly higher resistance to cefoxitin compared to other *bla*_CTX − M_ types. *E. coli* isolates that contain the *bla*_CTX − M−55_ gene frequently showed resistance to extended-spectrum cephalosporin, including cefoxitin (Hayer et al., 2022). Moreover, *bla*_CTX − M−1_ and *bla*_CTX − M−14_ carrying *E. coli* isolates demonstrated strong resistance to fluoroquinolones, consistent with previous findings (Silva et al., 2023). Furthermore, we found that *bla*_CTX − M_-carrying *E. coli* strains exhibited high levels of resistance to other non-β-lactam antimicrobials, including chloramphenicol, ciprofloxacin, nalidixic acid, streptomycin, and tetracycline. *E. coli* isolates from humans and food-producing animals typically exhibit co-resistance to cephalosporin and non-β-lactam antimicrobials (Balkhed et al., 2013; Seo et al., 2023).

Colistin is regarded as a last-resort antibiotic for treating multidrug-resistant bacterial infections. Nonetheless, the coexistence of the third-generation cephalosporin-resistant gene and colistin resistance further complicates the management and treatment of bacterial infections. In our investigation, colistin resistance was detected in *bla*_CTX − M_ isolates, including *bla*_CTX − M−55_-carrying *E. coli* strains. Our previous study identified colistin resistance in *bla*_CTX − M−55_-harboring *E. coli* isolated from pigs in South Korea (Ji-In et al., 2025). Moreover, prior research showed that plasmids carrying *bla*_CTX − M_ types of ESBL genes are commonly found in *mcr-1*-positive *E. coli* isolates (Shafiq et al., 2019). Especially, *bla*_CTX − M−55_-containing plasmids harbor the *mcr-1* gene in *E. coli* strains from livestock (Lupo et al., 2018) and humans (Ding et al., 2021). Plasmids containing multiple resistance genes are particularly concerning, as they facilitate horizontal gene transfer, which may lead to bacteria developing pan-drug resistance (Yoon et al., 2018).

We observed that the majority of *bla*_CTX − M−55_ and other *bla*_CTX − M_-carrying *E. coli* isolates exhibited MDR in this study. The increased MDR rate in *bla*_CTX − M_-harboring *E. coli* strains obtained from livestock, including cattle, pigs, and chickens, has been documented in China (Liu et al., 2022) and Korea (Song et al., 2020). Moreover, we found numerous MDR patterns in the cattle, pigs, and chickens isolates. The commonly identified MDR patterns in the *E. coli* isolated from these food-producing animals included resistance to chloramphenicol, ciprofloxacin, nalidixic acid, streptomycin, and tetracycline, which aligns with other studies (Silva et al., 2023). The prevalent occurrence of this resistance pattern may be attributed to the extensive use of these antimicrobials in Korean livestock [Animal and Plant Qurantine Agency (APQA), 2023]. In addition, the emergence of these antimicrobial resistances, as a main component of MDR in food animals, should be considered critically significant as MDR *E. coli* can be transferred to humans by direct contact with food-producing animals or their products via the food chain, presenting a significant risk to human health (Bennani et al., 2020).

The current investigation showed that 29.9% of ESBL-carrying *E. coli* demonstrated the capability to transfer β-lactamase genes. Variations in transferability were noted among *bla*_CTX − M_ types, with *bla*_CTX − M−1_ exhibiting a high transfer rate (68.8%), followed by *bla*_CTX − M−55_ (31.3%) and *bla*_CTX − M−14_ (29%) to the recipient *E. coli*. A prior study revealed that 38% of the ESBL-producing *E. coli* isolated from food animals were capable of being transmitted to the recipient (Ji-In et al., 2025). Furthermore, previous investigations showed that *bla*_CTX − M_ type genes, including *bla*_CTX − M−1_, *bla*_CTX − M−55_, and *bla*_CTX − M−14_, are highly transferable in ESBL-carrying *E. coli* isolated from livestock (Seo et al., 2023; Ji-In et al., 2025). Consequently, our findings indicate that ESBL-producing *E. coli* strains can spread these genes to other animals or humans.

In our investigation, the *bla*_CTX − M−55_-carrying transconjugants predominantly contain IncFIB, IncFIC, and IncF, while the *bla*_CTX − M−1_-harboring isolates contain IncI1. Prior research has revealed the presence of the IncFIB plasmid in *E. coli* isolates recovered from humans and livestock (Hounmanou et al., 2021). Moreover, IncF, IncI1, and IncFIC in *E. coli* isolates from humans and food-producing animals were investigated in different locations, including Australia (Puangseree et al., 2022) and Pakistan (Shafiq et al., 2022). The horizontal gene transfer of ESBL-harboring *E. coli*, often mediated by IncF, IncFIB, IncI1, and IncFIC plasmids, is a major driver of antimicrobial resistance dissemination among bacterial populations (Hounmanou et al., 2021).

The mobile genetic components play a crucial role in the development, interaction, and maintenance of antimicrobial resistance. They contribute to the spread of resistance by facilitating the mobilization and dispersion of genes that confer resistance to antimicrobials (Partridge et al., 2018). The majority of the transposable elements were found in Types I, II, and III, which are the most commonly identified genetic structures among the five types in this study. The major transposable element in Type I was IS*26*-IS*Ecp*1−*bla*_CTX − M−55_-*Orf477*, in Type II was IS*Ecp*1−*bla*_CTX − M−1_-*Orf477*, whereas IS*Ecp*1−*bla*_CTX − M−15_-*Orf477* and IS*Ecp*1−*bla*_CTX − M−14_*-*IS*903* were in Type III genetic structure. It was shown that downstream transposable elements IS*Ecp1* and *Orf477* are crucial for the activation and dissemination of *bla*_CTX − M_ genes (Jin-Tao et al., 2023). Furthermore, the transposable element IS*903* can promote the *bla*_CTX − M_ genes among the bacterial transmissible plasmids (Liao et al., 2015).

The MLST analysis revealed that ST752 was the most commonly found ST, followed by ST457, ST10, and ST1196. ST752 *E. coli* is predominantly detected in livestock and humans in different countries, including Korea (Park et al., 2023) and the UK (Sealey et al., 2024). Additionally, it was found that ST457, ST10, and ST1196 were linked to ESBL-encoding genes identified in humans and food-producing animals (Day et al., 2016; Lincopan et al., 2023). Furthermore, we found the STs varied according to *bla*_CTX − M_ types. In line with earlier research demonstrating that the STs of each *bla*_CTX − M_ type varied, the most common STs associated with *bla*_CTX − M−55_, *bla*_CTX − M−14_, *bla*_CTX − M−15_, and *bla*_CTX − M−1_ were ST457 and ST752 (Choi et al., 2014; Duggett et al., 2024). It was shown that STs in various *bla*_CTX − M_ types were identified in *E. coli* isolated from livestock (Al Mir, 2020). However, they possess the ability to infect humans, raising significant health hazards, which concurs with previous studies indicating that antimicrobial-resistant *E. coli* shares similarities with STs (Wang et al., 2013). Our phylogenetic analysis showed that numerous STs shared close genetic relationships with ST10. It was revealed that ST10 is a highly diverse phylogeny comprising several lineages clustering by geography or source of isolates (Reid et al., 2019). Moreover, ST10 *E. coli*, a major clonal group, has been identified in food-producing animals and is known to be frequently resistant to antimicrobials (Silva et al., 2023). Moreover, identifying common STs, such as ST752, ST457, and ST1196, suggests that certain successful clones might contribute significantly to the dissemination of resistance. Additionally, several STs were identified in only a single isolate, suggesting independent acquisition of third-generation cephalosporin resistance in these cases.

The NGS analysis revealed the presence of multiple antimicrobial resistance genes, including those for beta-lactams, aminoglycosides, sulfonamides, phenicols, tetracyclines, and colistin, corroborating their resistance to these agents. Among them, a range of resistance genes, such as *aph3”)-Ib, aph(6)-Id*, and *aadA22*, was detected, demonstrating that these genes can provide resistance to aminoglycosides in *E. coli* isolated from food animals (Joddha et al., 2023). Resistance to sulfonamide frequently results from the accumulation of *sul2* in *E. coli* isolates from humans and food-producing animals (Pokludová, 2020; Belina et al., 2024). Moreover, *tet(A)* and *floR*, which confer resistance to tetracycline and phenicol, respectively, were identified in *E. coli* isolated from chickens and pigs in many countries, including China (Zhou et al., 2022) and Brazil (Pissetti et al., 2017), consistent with our findings. The plasmids contain several metal resistance genes, such as *copA, zinC*, and *arsB*, which confer resistance to copper, zinc, and arsenic in *E. coli*. Furthermore, the presence of the *mcr-1* gene, along with other antimicrobial resistance genes, within a single plasmid underscores the need for coordinated management and control of resistant *E. coli* in both humans and animals.

The resistance of fluoroquinolone in *E. coli* resulted from mutations in the subunits comprising topoisomerase II (*gyrA* and *gyrB*) and IV (*parC* and *parE*). In this study, mutations in *gyrA* at S83L and D87Y, and in *parC* at S80I, and in *parE* at S458A were detected. *E. coli* exhibiting resistance to ciprofloxacin generally harbors at least two mutations in *gyrA*, accompanied by other mutations in QRDRs (Das et al., 2023). The additional mutations in the QRDRs constitute a crucial level of resistance to fluoroquinolones (Vanni et al., 2014). Moreover, the S458A mutation in *parE* has been described in *E. coli* isolated from food animals (Tang et al., 2019).

This investigation found the presence of a large number of virulence factors. Of them, factors involved in adhesion and colonization (*lpf* ) are crucial in triggering antimicrobial resistance in *E. coli* (Coppens et al., 2015). The genes (*pap*) encoding the pathogenicity island of uropathogenic *E. coli* strains are essential for forming intimate interactions with host cells for infections (Slavchev et al., 2013). Furthermore, the stress resistance genes (*sod*) augment the pathogenicity by associating with virulence in *E. coli*. Moreover, genes associated with toxin-antitoxin systems are helpful for the stability of mobile genetic elements and growth regulation in *E. coli* (Kedzierska and Hayes, 2016).

## 5 Conclusion

This 14-year longitudinal study (2010–2023) demonstrates a significant increase in third-generation cephalosporin resistance in *E. coli* from Korean livestock, particularly in chickens and pigs. A major genetic shift was observed since 2014, with *bla*_CTX − M−55_ replacing *bla*_CTX − M−14_ and *bla*_CTX − M−15_ as the predominant variant. The high prevalence of multidrug resistance (88.8%) and the emergence of isolates co-harboring *mcr-1* and ESBL genes on megaplasmids pose a severe threat to both veterinary and public health. The successful horizontal transfer of these resistance determinants, primarily mediated by IncF and IncI1 plasmids and mobile elements such as IS*Ecp1*, facilitates rapid dissemination of resistance across different clones (e.g., ST752, ST457, and ST10). Our findings underscore that livestock serves as a critical reservoir for highly transferable and virulent ESBL-producing *E. coli*. Consequently, strengthened antimicrobial stewardship and continuous “One Health” surveillance are imperative to mitigate the spread of these resistant lineages through the food chain.

## Data Availability

The original contributions presented in the study are included in the article/Supplementary material, further inquiries can be directed to the corresponding author.
