# Supplementary material for: Evolutionary trends and genetic determinants of third-generation cephalosporin resistance in Escherichia coli from Korean livestock: a 14-year longitudinal study, 2010–2023
Source: Front Microbiol. 2026 May 8;17:1816478. doi: 10.3389/fmicb.2026.1816478 (PMC13194429; doi:10.3389/fmicb.2026.1816478)
Supplement: Supplementary file 1 [file Table_1.DOCX]

***Supplementary Material***

Supplementary Table 1. List of primer sequences and polymerase chain reaction (PCR) conditions

| Primer | Sequence (5'-3') | Amplicon size (bp) | | PCR condition | References |
| --- | --- | --- | --- | --- | --- |
| CTX-M-Universal^a^ | ATGTGCAGYACCAGTAARGTKATGGC | 593 | | 35 cycles; 95℃ 1min + 60℃ 1min + 72℃ 1min | (Batchelor et al., 2005) |
|  | TGGGTRAARTARGTSACCAGAAYCAGCGG |  |  |  |  |
| CTX-M-1 families^a^ | AAGACTGGGTGTGGCATTGA | 670 | | 35 cycles; 95℃ 1min + 60℃ 1min + 72℃ 1min | (Batchelor et al., 2005) |
|  | AGGCTGGGTGAAGTAAGTGA |  |  |  |  |
| CTX-M-2 families^a^ | CTGGAAGCCCTGGAGAAAAG | 789 | 35 cycles; 95℃ 1min + 60℃ 1min + 72℃ 1min | | (Batchelor et al., 2005) |
|  | TACCTCGCTCCATTTATTGC |  |  |  |  |
| CTX-M-8 families^a^ | GCCTGTATTTCGCTGTTG | 686 | 35 cycles; 95℃ 1min + 60℃ 1min + 72℃ 1min | | (Batchelor et al., 2005) |
|  | TGTCATTCGTCGTACCATAA |  |  |  |  |
| CTX-M-9 families^a^ | GCTTTATGCGCAGACGAGTG | 703 | 35 cycles; 95℃ 1min + 60℃ 1min + 72℃ 1min | | (Batchelor et al., 2005) |
|  | GCCAGATCACCGCAATATCA |  |  |  |  |
| CTX-M-1 sequencing^a^ | CAGCACTTTTGCCGTCTAAG | 1130 | 35 cycles; 94℃ 30 sec + 55℃ 30 sec + 72℃ 1min | | (Yaita et al., 2014) |
|  | AAAAATGATTGAAAGGTGGT |  |  |  |  |
| CTX-M-9 sequencing^a^ | GAAGCAGTCTAAATTCTTCGTGAAATAG | 1100 | 35 cycles; 94℃ 30 sec + 60℃ 30 sec + 72℃ 1min | | (Yaita et al., 2014) |
|  | GGGCCAGTTGGTGATTTTGA |  |  |  |  |
| *ISEcp1^b^* | CGAGAGAAATTACACCGGTCA | 1547 | 35 cycles; 95℃ 30sec + 53℃ 30sec + 72℃ 1min | | (Eckert et al., 2006) |
|  | AGCCCTTCAATGCTGATGTC |  |  |  |  |
| *Orf477 ^b^* | TGTATGCGATGTCTGAACTG | 1238 | 35 cycles; 95℃ 30sec + 50℃ 30sec + 72℃ 1min | | (Saladin et al., 2002) |
|  | CTCAATAGCAGCTCGGAATA |  |  |  |  |
| *IS903 ^b^* | TACCGAGCTGTTCCTTGTGG | 836 | 35 cycles; 95℃ 30sec + 53℃ 30sec + 72℃ 1min | | (Tamang et al., 2014) |
|  | CGGTTGTAATCTGTTGTCCA |  |  |  |  |
| *IS26 ^b^* | CTGCTTTACCAACAACATTCC | 618 | 35 cycles; 95℃ 30sec + 56℃ 30sec + 72℃ 1min | | (Eckert et al., 2006) |
|  | TTGTCCGGGTTGTACTCGTC |  |  |  |  |
| *IncI1* | CGAAAGCCGGACGGCAGAA | 139 | 30 cycles; 94℃ 30sec + 60℃ 30sec + 72℃ 90sec | | (Johnson et al., 2007) |
|  | TCGTCGTTCCGCCAAGTTCGT |  |  |  |  |
| *IncF* | CTATGGCCCTGCAAACGCGCCAGAAA | 534 | 30 cycles; 94℃ 30sec + 60℃ 30sec + 72℃ 90sec | | (Johnson et al., 2007) |
|  | TCACGCGCCAGGGCGCAGCC |  |  |  |  |
| *IncN* | GTCTAACGAGCTTACCGAAG | 559 | 30 cycles; 94℃ 30sec + 60℃ 30sec + 72℃ 90sec | | (Johnson et al., 2007) |
|  | GTTTCAACTCTGCCAAGTTC |  |  |  |  |
| *IncFIA* | CCATGCTGGTTCTAGAGAAGGTG | 462 | 30 cycles; 94℃ 30sec + 60℃ 30sec + 72℃ 90sec | | (Johnson et al., 2007) |
|  | GTATATCCTTACTGGCTTCCGCAG |  |  |  |  |
| *IncFIB* | GGAGTTCTGACACACGATTTTCTG | 702 | 30 cycles; 94℃ 30sec + 60℃ 30sec + 72℃ 90sec | | (Johnson et al., 2007) |
|  | CTCCCGTCGCTTCAGGGCATT |  |  |  |  |
| *IncFIC* | GTGAACTGGCAGATGAGGAAGG | 262 | 30 cycles; 94℃ 30sec + 60℃ 30sec + 72℃ 90sec | | (Johnson et al., 2007) |
|  | TTCTCCTCGTCGCCAAACTAGAT |  |  |  |  |
| *IncB/O* | GCGGTCCGGAAAGCCAGAAAAC | 159 | 30 cycles; 94℃ 30sec + 60℃ 30sec + 72℃ 90sec | | (Johnson et al., 2007) |
|  | TCTGCGTTCCGCCAAGTTCGA |  |  |  |  |
| *IncHI2* | TTTCTCCTGAGTCACCTGTTAACAC | 644 | 30 cycles; 94℃ 30sec + 60℃ 30sec + 72℃ 90sec | | (Johnson et al., 2007) |
|  | GGCTCACTACCGTTGTCATCCT |  |  |  |  |
| *IncK2* | GCGGTCCGGAAAGCCAGAAAAC | 160 | 30 cycles; 94℃ 30sec + 60℃ 30sec + 72℃ 90sec | | (Johnson et al., 2007) |
|  | TCTTTCACGAGCCCGCCAAA |  |  |  |  |

^a^ Primer sequences used in *bla*_CTX-M_

^b^ Primer sequences used in determination of genetic environment of *bla*_CTX-M_

**References**

Batchelor, M., Threlfall, E. J., and Liebana, E. (2005). Cephalosporin resistance among animal-associated *Enterobacteria*: a current perspective. *Expert Rev. Anti. Infect. Ther.* 3, 403–417. doi: 10.1586/14787210.3.3.403

Eckert, C., Gautier, V., and Arlet, G. (2006). DNA sequence analysis of the genetic environment of various *bla*_CTX-M_ genes. *J. Antimicrob. Chemother.* 57, 14–23. https://doi.org/10.1093/jac/dki398

Johnson, T. J., Wannemuehler, Y. M., Johnson, S. J., Logue, C. M., White, D. G., Doetkott, C., et al. (2007). Plasmid replicon typing of commensal and pathogenic *Escherichia coli* isolates. *Appl. Environ. Microbiol.* 73, 1976–1983. https://doi.org/10.1128/AEM.02171-06

Saladin, M., Cao, V. T. B., Lambert, T., Donay, J.-L., Herrmann, J.-L., Ould-Hocine, Z., et al. (2002). Diversity of CTX-M β-lactamases and their promoter regions from *Enterobacteriaceae* isolated in three Parisian hospitals. *FEMS Microbiol. Lett.* 209, 161–168. https://doi.org/ 10.1111/j.1574-6968.2002.tb11126.x

Tamang, M. D., Gurung, M., Kang, M.-S., Nam, H.-M., Moon, D. C., Jang, G.-C., et al. (2014). Characterization of plasmids encoding CTX-M β-lactamase and their addiction systems in *Escherichia coli* isolates from animals. *Vet. Microbiol.* 174, 456–462. https://doi.org/ 10.1016/j.vetmic.2014.10.004

Yaita, K., Aoki, K., Suzuki, T., Nakaharai, K., Yoshimura, Y., Harada, S., et al. (2014). Epidemiology of extended-spectrum β-lactamase producing *Escherichia coli* in the stools of returning Japanese travelers, and the risk factors for colonization. *PLoS One* 9, e98000. https://doi.org/ 10.1371/journal.pone.0098000

Supplementary Table 2. Distribution of ESBL genes in *Escherichia coli* isolated from cattle during 2010–2023 in South Korea

| Year | No. of gene | *bla*_CTX-M-14_ | *bla*_CTX-M-15_ | *bla*_CTX-M-55_ | *bla*_CTX-M-65_ |
| --- | --- | --- | --- | --- | --- |
| 2011 | 0 | – | – | – | – |
| 2012 | 1 | 1 | – | – | – |
| 2013 | 0 | – | – | – | – |
| 2014 | 1 | – | – | 1 | – |
| 2015 | 4 | – | 2 | 1 | 1 |
| 2016 | 1 | – | – | 1 |  |
| 2017 | 0 | – | – | – | – |
| 2018 | 0 | – | – | – | – |
| 2019 | 2 | – | 1 | 1 | – |
| 2020 | 0 | – | – | – | – |
| 2021 | 1 | – | – | 1 | – |
| 2022 | 1 | – | – | 1 | – |
| 2023 | 1 | 1 | – | – | – |
| Total | 12 | 2 (16.7) | 3 (25.0) | 6 (50.0) | 1 (8.3) |

Supplementary Table 3. Distribution of ESBL genes in *Escherichia coli* isolated from pigs during 2010–2023 in South Korea

| Year | No. of gene | *bla*_CTX-M-1_ | *bla*_CTX-M-3_ | *bla*_CTX-M-14_ | *bla*_CTX-M-15_ | *bla*_CTX-M-27_ | *bla*_CTX-M-55_ | *bla*_CTX-M-65_ | *bla*_CTX-M-101_ |
| --- | --- | --- | --- | --- | --- | --- | --- | --- | --- |
| 2011 | 0 | – | – | – | – | – | – | – | – |
| 2012 | 0 | – | – | – | – | – | – | – | – |
| 2013 | 1 | – | – | 1 | – | – | – | – | – |
| 2014 | 3 | – | – | – | – | – | 3 | – | – |
| 2015 | 20 | 1 | – | 1 | 6 | 1 | 10 | 1 | – |
| 2016 | 8 | – | 2 | – | – | – | 6 | – | – |
| 2017 | 6 | – | – | 1 | – | – | 4 | 1 | – |
| 2018 | 4 | – | – | – | – | – | 3 | 1 | – |
| 2019 | 8 | – | – | 3 | – | – | 5 | – | – |
| 2020 | 6 | – | – | – | 1 | – | 5 | – | – |
| 2021 | 10 | – | – | – | 3 | – | 7 | – | – |
| 2022 | 6 | – | 1 | – | 1 | – | 4 | – | – |
| 2023 | 9 | – | – | 3 | 2 | 1 | 2 | – | 1 |
| Total | 81 | 1 (1.2) | 3 (3.7) | 9 (11.1) | 13 (16.0) | 2 (2.5) | 49 (60.5) | 3 (3.7) | 1 (1.2) |

Supplementary Table 4. Distribution of ESBL genes in *Escherichia coli* isolated from chickens during 2010–2023 in South Korea

| Year | No. of gene | *bla*_CTX-M-1_ | *bla*_CTX-M-3_ | *bla*_CTX-M-8_ | *bla*_CTX-M-14_ | *bla*_CTX-M-15_ | *bla*_CTX-M-27_ | *bla*_CTX-M-55_ | *bla*_CTX-M-65_ |
| --- | --- | --- | --- | --- | --- | --- | --- | --- | --- |
| 2011 | 5 | – | – | – | 1 | 4 | – | – | – |
| 2012 | 4 | 1 | – | – | – | 3 | – | – | – |
| 2013 | 5 | – | – | – | 2 | 2 | – | – | 1 |
| 2014 | 6 | 1 | – | – | 1 | – | – | 3 | 1 |
| 2015 | 9 | 2 | – | – | 2 | – | – | 6 | 2 |
| 2016 | 11 | 4 | – | – | 2 | – | – | 7 | – |
| 2017 | 8 | 3 | 1 | – | 2 | 1 | – | 1 | – |
| 2018 | 14 | – | – | – | 4 | – | 2 | 7 | 1 |
| 2019 | 11 | 1 | – | 1 | 2 | – | – | 7 | 1 |
| 2020 | 10 | 2 | – | – | 2 | – | – | 5 | 1 |
| 2021 | 7 | – | – | – | 2 | - | – | 5 | -– |
| 2022 | 9 | 1 | – | – | – | 1 | – | 7 | 1 |
| 2023 | 5 | – | – | – | – | 1 | 1 | 4 | – |
| Total | 112 | 15 (13.4) | 1 (0.9) | 1 (0.9) | 20 (17.9) | 12 (10.7) | 3 (2.7) | 52 (46.4) | 8 (7.1) |

Supplementary Table 5. Antimicrobial resistance patterns of *Escherichia coli* isolated from cattle, pigs, and chickens during 2010–2023 in South Korea

| *bla*_CTX-M_ type | No. of isolate | Resistance patterns (No. of isolates) |
| --- | --- | --- |
| *bla*_CTX-M-1_ | 16 | CIP NAL (n=1), CIP NAL TET (n=3), CIP NAL STR TET (n=2), CHL NAL STR TET (n=1), CHL STR TET SXT (n=1), CIP NAL TET SXT (n=1), GEN NAL STR TET (n=1), CHL CIP NAL STR TET (n=1), CHL CIP NAL STR TET SXT (n=2), CHL CIP COL NAL STR TET (n=1), CHL CIP GEN NAL STR TET (n=1), CHL CIP GEN NAL STR TET SXT (n=1) |
| *bla*_CTX-M-3_ | 4 | CHL STR TET (n=1), STR TET SXT (n=1), CHL NAL STR TET SXT (n=1), CHL GEN NAL STR TET SXT (n=1) |
| *bla*_CTX-M-8_ | 1 | CHL (n=1) |
| *bla*_CTX-M-14_ | 31 | STR (n=1), CIP COL (n=2), CIP NAL (n=1), NAL TET (n=1), CIP COL SXT (n=1), STR TET SXT (n=1), CHL CIP GEN NAL (n=2), CHL CIP NAL SXT (n=1), CHL GEN NAL STR (n=1), CHL NAL STR TET (n=1), CIP GEN NAL STR (n=1), CHL CIP GEN NAL STR (n=2), CHL CIP GEN NAL SXT (n=2), CHL GEN NAL STR TET (n=2), CHL CIP NAL STR TET (n=1), CHL GEN STR TET SXT (n=1), CIP GEN NAL TET SXT (n=1), CIP NAL STR TET SXT (n=1), CHL CIP COL GEN NAL TET (n=1), CHL CIP GEN NAL STR SXT (n=1), CHL CIP GEN NAL TET SXT (n=1), CHL CIP GEN NAL STR TET SXT (n=4) |
| *bla*_CTX-M-15_ | 28 | CIP NAL (n=2), STR TET (n=2), NAL STR TET (n=2), CIP COL SXT (n=1), CHL NAL STR TET (n=1), CIP COL STR SXT (n=1), CIP NAL STR SXT (n=1), COL GEN STR TET (n=1), NAL STR TET SXT (n=1), CHL CIP NAL TET SXT (n=1), CHL COL STR TET SXT (n=1), CHL GEN NAL STR TET (n=1), CIP GEN NAL STR TET (n=1), COL GEN NAL STR TET (n=1), CHL CIP NAL STR TET SXT (n=1), CIP GEN NAL STR TET SXT (n=1), CHL CIP GEN NAL STR TET SXT (n=5), CIP COL GEN NAL STR TET SXT (n=1), CHL CIP COL GEN NAL STR TET SXT (n=1) |
| *bla*_CTX-M-27_ | 5 | CIP NAL (n=1), CIP COL SXT (n=1), CHL CIP NAL TET (n=1), CHL STR TET SXT (n=1), CHL CIP GEN NAL STR TET SXT (n=1) |
| *bla*_CTX-M-55_ | 107 | NAL (n=3), STR (n=1), CIP NAL (n=3), CHL GEN (n=1), CIP COL (n=1), GEN STR (n=1), NAL TET (n=1), STR TET (n=1), TET SXT (n=1), CHL CIP NAL (n=4), CHL NAL STR (n=4), CIP NAL STR (n=3), CIP NAL TET (n=3), CHL STR TET (n=2), CHL TET SXT (n=2), CHL NAL TET (n=1), CHL STR SXT (n=1), CIP COL SXT (n=1), CIP GEN NAL (n=1), CHL STR TET SXT (n=8), CHL CIP NAL TET (n=2), CHL NAL STR TET (n=2), CIP GEN NAL TET (n=2), CIP NAL STR SXT (n=2), CHL CIP NAL STR (n=1), CHL COL NAL STR (n=1), CHL GEN STR TET (n=1), CHL GEN TET SXT (n=1), CHL NAL STR SXT (n=1), CHL NAL TET SXT (n=1), CHL CIP NAL STR TET (n=11), CHL CIP NAL TET SXT (n=5), CHL GEN STR TET SXT (n=3), CHL CIP GEN NAL SXT (n=2), CHL CIP GEN NAL STR (n=1), CHL CIP GEN NAL TET (n=1), CHL CIP NAL STR SXT (n=1), CHL GEN NAL STR SXT (n=1), CHL GEN NAL TET SXT (n=1), CHL NAL STR TET SXT (n=1), CIP GEN NAL STR TET (n=1), CIP NAL STR TET SXT (n=1), CHL CIP NAL STR TET SXT (n=8), CHL CIP GEN NAL STR TET (n=5), CHL CIP COL NAL STR TET (n=1), CHL CIP GEN NAL STR SXT (n=1), CHL CIP GEN NAL TET SXT (n=1), CHL CIP GEN NAL STR TET SXT (n=3), CHL CIP COL NAL STR TET SXT (n=1) |
| *bla*_CTX-M-65_ | 12 | CHL NAL STR (n=1), CHL CIP NAL SXT (n=1), CHL NAL STR TET (n=1), GEN NAL STR TET (n=1), CHL CIP GEN NAL SXT (n=2), CHL GEN STR TET SXT (n=1), CHL CIP NAL STR TET SXT (n=3), CHL CIP GEN NAL STR TET SXT (n=2) |
| *bla*_CTX-M-101_ | 1 | CIP COL SXT (n=1) |

CHL, chloramphenicol; CIP, ciprofloxacin; COL, colistin; GEN, gentamicin; NAL, nalidixic acid; STR, streptomycin; TET, tetracycline; SXT, trimethoprim/sulfamethoxazole.

Supplementary Table 6. Multi-locus sequence typing (MLST) profiles of ESBL-harboring *Escherichia coli* isolated from livestock during 2010–2023 in South Korea

| STs | No. of isolates (%) | *bla*_CTX-M-55_  (n = 112) | *bla*_CTX-M-15_  (n = 32) | *bla*_CTX-M-14_  (n = 31) | *bla*_CTX-M-1_  (n = 16) | *bla*_CTX-M-65_  (n = 12) | *bla*_CTX-M-27_  (n = 5) | *bla*_CTX-M-3_  (n = 4) | *bla*_CTX-M-8_  (n = 1) | *bla*_CTX-M-101_  (n = 1) |
| --- | --- | --- | --- | --- | --- | --- | --- | --- | --- | --- |
| ST 752 | 14 (6.5) | 4 | - | 5 | 1 | 3 | 1 | - | - | - |
| ST 457 | 11 (5.1) | 9 | 2 | - | - | - | - | - | - | - |
| ST 10 | 10 (4.7) | 6 | - | - | 2 | - | - | 1 | 1 | - |
| ST 1196 | 10 (4.7) | 5 | - | 3 | 2 | - | - | - | - | - |
| ST 155 | 8 (3.7) | 3 | 2 | - | 2 | 1 | - | - | - | - |
| ST 93 | 7 (3.3) | 3 | - | 2 | 2 | - | - | - | - | - |
| ST 101 | 7 (3.3) | 6 | 1 | - | - | - | - | - | - | - |
| ST 2170 | 7 (3.3) | 7 | - | - | - | - | - | - | - | - |
| ST 58 | 6 (2.8) | 3 | - | 1 | - | - | 1 | - | - | 1 |
| ST 410 | 5 (2.3) | 4 | 1 | - | - | - | - | - | - | - |
| ST 69 | 4 (1.9) | 3 | 1 | - | - | - | - | - | - | - |
| ST 117 | 4 (1.9) | 1 | 3 | - | - | - | - | - | - | - |
| ST 9405 | 4 (1.9) | 4 | - | - | - | - | - | - | - | - |
| ST 75 | 3 (1.4) | 3 | - | - | - | - | - | - | - | - |
| ST 88 | 3 (1.4) | 1 | - | 1 | - | 1 | - | - | - | - |
| ST 602 | 3 (1.4) | - | 2 | - | - | 1 | - | - | - | - |
| ST 767 | 3 (1.4) | 3 | - | - | - | - | - | - | - | - |
| ST 1642 | 3 (1.4) | 1 | 2 | - | - | - | - | - | - | - |
| ST 2179 | 3 (1.4) | - | - | - | - | 3 | - | - | - | - |
| ST 2453 | 3 (1.4) | - | - | 3 | - | - | - | - | - | - |
| ST 3285 | 3 (1.4) | 3 | - | - | - | - | - | - | - | - |
| ST 5899 | 3 (1.4) | 1 | 1 | 1 | - | - | - | - | - | - |
| ST 70 | 2 (0.9) | 2 | - | - | - | - | - | - | - | - |
| ST 131 | 2 (0.9) | 1 | 1 | - | - | - | - | - | - | - |
| ST 156 | 2 (0.9) | - | 1 | - | - | - | - | 1 | - | - |
| ST 162 | 2 (0.9) | - | - | 1 | 1 | - | - | - | - | - |
| ST 224 | 2 (0.9) | 1 | 1 | - | - | - | - | - | - | - |
| ST 297 | 2 (0.9) | - | - | 2 | - | - | - | - | - | - |
| ST 2505 | 2 (0.9) | 2 | - | - | - | - | - | - | - | - |
| ST 3941 | 2 (0.9) | 2 | - | - | - | - | - | - | - | - |
| ST 48 | 1 (0.5) | 1 | - | - | - | - | - | - | - | - |
| ST 57 | 1 (0.5) | - | 1 | - | - | - | - | - | - | - |
| ST 165 | 1 (0.5) | - | - | - | - | - | 1 | - | - | - |
| ST 206 | 1 (0.5) | - | - | - | - | - | 1 | - | - | - |
| ST 212 | 1 (0.5) | 1 | - | - | - | - | - | - | - | - |
| ST 345 | 1 (0.5) | 1 | - | - | - | - | - | - | - | - |
| ST 349 | 1 (0.5) | - | - | - | 1 | - | - | - | - | - |
| ST 354 | 1 (0.5) | - | - | 1 | - | - | - | - | - | - |
| ST 453 | 1 (0.5) | - | 1 | - | - | - | - | - | - | - |
| ST 603 | 1 (0.5) | 1 | - | - | - | - | - | - | - | - |
| ST 641 | 1 (0.5) | 1 | - | - | - | - | - | - | - | - |
| ST 654 | 1 (0.5) | 1 | - | - | - | - | - | - | - | - |
| ST 744 | 1 (0.5) | 1 | - | - | - | - | - | - | - | - |
| ST 993 | 1 (0.5) | - | 1 | - | - | - | - | - | - | - |
| ST 1112 | 1 (0.5) | 1 | - | - | - | - | - | - | - | - |
| ST 1163 | 1 (0.5) | - | 1 | - | - | - | - | - | - | - |
| ST 1431 | 1 (0.5) | - | 1 | - | - | - | - | - | - | - |
| ST 1485 | 1 (0.5) | - | - | - | - | - | - | 1 | - | - |
| ST 1684 | 1 (0.5) | - | - | - | - | - | - | 1 | - | - |
| ST 1725 | 1 (0.5) | - | - | 1 | - | - | - | - | - | - |
| ST 2040 | 1 (0.5) | 1 | - | - | - | - | - | - | - | - |
| ST 2309 | 1 (0.5) | - | 1 | - | - | - | - | - | - | - |
| ST 2628 | 1 (0.5) | 1 | - | - | - | - | - | - | - | - |
| ST 2705 | 1 (0.5) | 1 | - | - | - | - | - | - | - | - |
| ST 2732 | 1 (0.5) | 1 | - | - | - | - | - | - | - | - |
| ST 2847 | 1 (0.5) | - | - | - | - | 1 | - | - | - | - |
| ST 4429 | 1 (0.5) | 1 | - | - | - | - | - | - | - | - |
| ST 5229 | 1 (0.5) | - | - | 1 | - | - | - | - | - | - |
| ST 6096 | 1 (0.5) | 1 | - | - | - | - | - | - | - | - |
| ST 6731 | 1 (0.5) | - | 1 | - | - | - | - | - | - | - |
| ST 6817 | 1 (0.5) | 1 | - | - | - | - | - | - | - | - |
| ST 7584 | 1 (0.5) | - | - | - | - | - | 1 | - | - | - |
| ST 8165 | 1 (0.5) | - | - | 1 | - | - | - | - | - | - |
| ST 14922 | 1 (0.5) | 1 | - | - | - | - | - | - | - | - |
| NT | 40 (18.7) | 18 | 7 | 8 | 5 | 2 | - | - | - | - |
| Total | 214 | 112 | 32 | 31 | 16 | 12 | 5 | 4 | 1 | 1 |

NT, not tested. -, not detected.

Supplementary Table 7. Next-generation sequencing analysis of *Escherichia coli* isolated from livestock during 2010–2023 in South Korea

| Isolate | Animal | Plasmid ID | Resistance pattern | Conjuga-tion | Transfer pattern | Replicon type | Position | Size (bp) | No. of CDS | p. mutation | Resistance gene | Metal resistance | Mobile genetic elements (MGEs) | Virulence factor | Replication/  maintenance | Toxin/  antitoxin |
| --- | --- | --- | --- | --- | --- | --- | --- | --- | --- | --- | --- | --- | --- | --- | --- | --- |
| V15-19-A02-002-011 | Healthy pig | pA269 | AMP CTX CHL STR NAL COL | O | AMP CTX COL | – | Chromos-ome | 5,086,306 | 4,779 | – | *fosA7* | *copA,cueR,cueO,cusA,cusB,cusC,cusF,cusR,cusS,zntA,zntR,zitB,znuA,znuB,znuC,mntH,mntB,mntR,mntS,feoA,feoB,feoC,corA,rcnA,rcnB,rcnR* | *ISEc12,ISPpu7,isec17,IS609,ISEc20,ISEsa1,ISEc5,ISEc1,ISCfr6,ISYps3,IS1R,IS1D,IS682,ISEc13,IS4,IS200C,ISEc40,ISEc16,IS3,IS621,ISSen4,IS1222,IS630,ISVsa17,IS911,ISLad1,ISEc18,ISEc53,ISVsa5,ISSen7,intA,intQ,intS,xerC,xerD,tnpR,hin* | *fimA,fimC,fimD,fimF,fimG,fimH,focC,sfaA,sfaG,sfaS,sfaH,papA,papB,papC,papD,papE,papG,papH,papK,lpfA,lpfB,lpfD,lpfE,ecpR,ecpA,ecpB,ecpC,ecpD,ecpE,csgA,csgB,csgC,csgD,csgE,csgF,csgG,pgaA,pgaB,pgaC,pgaD,bcsA,bcsB,bcsC,bcsZ,bcsQ,bcsE,bcsG,entA,entB,entC,entD,entE,entF,entH,entS,fepA,fepB,fepC,fepD,fepG,fepE,fes,chuR,ompA,ompC,ompF,ompD,ompX,ompG,ompN,lamB,pagN,hlyE,pic,spaO,spaP,spaQ,spaR,spaS,prgH,prgK,orgA* | *dnaA,dnaB,dnaC,dnaE,dnaG,dnaN,recA,recB,recC,recD,recF,recG,recN,recO,uvrA,uvrB,uvrC,uvrD,mutS,mutL,mutH,ssb,gyrA,gyrB,topA,parC,parE,mukB,mukE,mukF,ftsZ,ftsA,ftsQ,ftsW,ftsI,ftsL,ftsK,ftsN,zipA* | *mazE,mazF,relB,relE,yefM,yoeB,hipA,hipB,vapB,vapC,prlF,yhaV,higA,higB-2,hokC,hokE,ghoT,ghoS,tisB,ratA,cbtA,cbeA,parD1,hicB* |
|  |  |  |  |  |  | IncFIB, IncFII | Plasmid 1 | 109,427 | 122 | – | *bla*_CTX-M-55_, *lnu(F), aadA22,floR* | *mntB* | *xerC, IS629, ISEcp1, IS26, tnpR, Tn3, traI, traD, traQ, traN, traC, traV, traA, traY, traJ, traM, ssb* | *ompT, cvaC, cma, cmi, aidA, iutA, iucD, iucC, iucB, iucA* | *repB, cai, pifC, finO, ylpA, psiB, sopB, crcB, eno, umuC* | *vapC, pemK, pemI, pndA, vapB* |
|  |  |  |  |  |  | IncI1 | Plasmid 2 | 96,180 | 102 | – | *aph(3'')-Ib, aph(6)-Id, bla*_TEM-1_*, FloR, sul2* | – | *ssb,ISRor2,Tn2,tnpR* | *hdfR, rfaH* | *repA,umuC,umuD,yhdJ,klcA,noc,psiB* | *pndA* |
|  |  |  |  |  |  | IncI2 | Plasmid 3 | 72,641 | 88 | – | *mcr-1.1* | *zinC* | *topB, traL, ISVsa5, ISSbo1, ISSen6, rcbA, traG, xerC, mbeC* | *ymoA, proQ, relE, virB1, virB4, virB8, sctC, cna* | *dnaJ, flmC, repA, qmcA, ybbJ, rop, pap2* | – |
|  |  |  |  |  |  | rep_cluster_2350 | Plasmid 4 | 6,412 | 6 | – | – | – | *mbeA, mbeC* | *cna* | *rop* | – |
| V09-21-A03-002-012 | Healthy chicken | pA3176 | AMP CTX CHL TET STR CIP NAL COL | O | AMP CTX CHL STR COL | – | Chromosome | 5,347,414 | 4,964 | *gyrA* p.S83L *gyrA* p.D87N *parC* p.S80I *parE* p.S458A | *aph(6)-Id,aph(3'')-Ib,FloR,sul2,tet(A)* | *arsD,arsA,arsB,arsC,copA,cusC,cusB,cusA,cusF,zntA,zntR,mntH,mntB,mntS,mntR,znuA,znuB,znuC* | *ISEc31,ISPpu7,ISSso4,IS200C,ISKpn26,ISLad1,IS629,IS1414,IS2,IS1R,ISSen7,IS4,IS1397,IS421,ISEc39,ISKox3,ISSoEn2,ISSaen1,IS630,ISSen1,ISEc22,ISKpn38,ISEc21,IS100kyp,ISCro1,ISEc16,ISEc23,ISAs9,ISEc12,IS1203,IS3411,IS1A,IS609,IS911,ISSd1,IS30,IS1F,ISEc8,IS5,ISCep1,ISRor2,ISEc1,ISEc46,ISEc38,ISEc78,IS3,ISVsa3* | *fimA,fimC,fimD,fimF,fimG,fimH,fimI,lpfA,lpfD,ecpA,ecpB,ecpC,ecpD,ecpE,ecpR,sfmA,sfmC,sfmD,sfmH,sfmF,csgA,csgB,csgC,csgD,csgE,csgF,csgG,hlyE,pagN,icsA,sodC,sodC1* | *rep* | *cbeA,cbtA,higA,higB,higB2,hipA,hipB,yefM,yoeB,ghoT,ghoS,mqsR,mqsA,hicA,hicB,symE* |
|  |  |  |  |  |  | IncHI2A | Plasmid 1 | 101,997 | 101 | – | – | – | *traC* | *stiP, yceD, hha* | *uvrD,parM,repB,dam* | *higB-1* |
|  |  |  |  |  |  | rep_cluster_1704 | Plasmid 2 | 92,781 | 120 | – | – | – | *IS2* | *ybaQ* | *smc,dut,umuD,pphA,clpX,rdgC,parM,repE* | *hicB,higA,higB-1* |
|  |  |  |  |  |  | IncFII | Plasmid 3 | 92,557 | 109 | – | *floR, tet(A), aph(6)-Id, aph(3'')-Ib, sul2, fosA3,* *bla*_TEM_, *bla*_CTX-M-55_ | – | *IS1D, TN3, IS26, IS5075, ISPa38, ssb, traM, traJ, traA, traV, traC, traN, traQ, traS, traD, traI* | *hdfR* | *stbB, parM, yhdJ, klcA, noc, psiB, flmC, ylpA, finO* | *pemI, pemK, pndA* |
|  |  |  |  |  |  | IncI2 | Plasmid 4 | 68,620 | 87 | – | *mcr-1.1* | *zinC* | *IS609, traL, ISApl1, topB, xerC, traG, rcbA* | *proQ, ymoA, sctC, virB8, virB4, virB1, relE* | *repA, flmC, dnaJ, ybbJ, qmcA, pap2* | – |
|  |  |  |  |  |  | IncI1 | Plasmid 5 | 63,119 | 70 | – | – | – | *ISRor2, xerC* | *pld,yggR,ais,tcpT,rfaH* | *psiB, repA* | *pndA* |
|  |  |  |  |  |  | rep_cluster_2350 | Plasmid 6 | 12,766 | 13 | – | – | – | *mbeC,mbeA,ISPpu7,ISSso4,IS5* | – | *rop* | *cea* |
|  |  |  |  |  |  | rep_cluster_2370 | Plasmid 7 | 4,315 | 4 | – | – | – | – | – | *hindIIIM* | – |
|  |  |  |  |  |  | Col156 | Plasmid 8 | 4,234 | 3 | – | – | – | – | – | – | – |
| V06-15-A03-002-010 | Healthy chicken | pA357 | AMP CTX CHL TET STR CIP NAL COL | O | AMP CTX COL | – | Chromosome | 4,944,865 | 4,581 | *gyrA* p.S83L *gyrA* p.D87N *parC* p.S80I | *aph(6)-Id, aph(3’’)-Ib, FloR, sul2, tet(A)* | *arsB,arsC,chrR,copA,cusA,cusB,cusC,cusF,cusR,cusS,rcnA,rcnB,rcnR,zinT,zntA,zntB,zntR* | *IS1A,IS1H,IS3,IS421,IS609,IS621,ISCep1,ISEc1,ISEc17,ISEc26,ISEc38,ISEc5,ISKpn26,ISRor2,ISSen7,ISVsa17,ISVsa3,ISYps3,intA,intS,xerC,xerD* | *chuR,clpV1,csgA,csgB,csgC,csgD,csgE,csgF,csgG,ecpA,ecpB,ecpC,ecpD,ecpE,ecpR,elfA,elfC,elfD,elfG,fimA,fimC,fimD,fimF,fimG,fimH,focC,hcp,hilA,hlyE,lpfA,lpfB,lpfD,lpfE,ompD,orgA,pagN,papA,papB,papC,papD,papF,papH,prgH,prgK,sfmA,sfmC,sfmD,sfmF,sfmH,sicA,spaO,spaP,spaQ,spaR,spaS,vgrG1* | *dnaA,dnaB,dnaC,dnaE,dnaG,dnaQ,dnaT,dnaX,ftsA,ftsB,ftsI,ftsL,ftsN,ftsQ,ftsW,ftsZ,gyrA,gyrB,hda,mukB,mukE,mukF,parC,parE,rep,seqA,topA,zipA* | *chpB,chpS,ghoS,ghoT,higA,higB-2,hipA,hipB,hokE,mazE,mazF,vapB,vapC,yefM,yoeB* |
|  |  |  |  |  |  | IncFIA,FIB,FIC | Plasmid 1 | 205,894 | 220 | – | – | *mntB, tdeA, yciC* | *xerC,hin,TnAs1,traI,traD,traQ,traN,traC,traV,traA,traY,traJ,traM,ssb,IS1351,ISCro3,ISLad2,ISBcen27,IS2,ISKpn28,IS3,ISEc17,ISEc8,IS30,ISEc37,IS1663,ISYps3,ISKpn26,IS100kyp,IS1A,ISSbo1,ISKpn42,IS421,IS1R,traR,xerD* | *ompT,fecE,relE,aidA,iutA,iucD,iucC,iucB,iucA,fes,pfeA,hbp,hmuU,proQ* | *repB,finO,ylpA,flmC,psiB,noc,klcA,sopB,crcB,eno,umuC* | *pndA,vapB,vapC,imm,col,mcjD,mcjA,ccdB,ccdA* |
|  |  |  |  |  |  | IncI1 | Plasmid 2 | 107,042 | 117 | – | *tet(A), sul2,* *bla*_CTX-M-1_ | – | *ISSbo1, IS26, xerC, ssb, ISSen7, ISCro1, xerC, ISEcp1* | *glmM, pld, yggR, ais, rfaH* | *repA, dinG, parM, umuC, umuD, yhdJ, klcA, noc, psiB* | *cia, pndA* |
|  |  |  |  |  |  | IncI2 | Plasmid 3 | 62,179 | 77 | – | *mcr-1.1* | *zinC* | *IS103, IS150, traL, mcr-1.1, topB, xerC, traG, rcbA* | *proQ, ymoA, sctC, virB8, virB4, virB1, relE* | *repA,flmC,ybbJ,qmcA,pap2* | – |
|  |  |  |  |  |  | rep_cluster_2335 | Plasmid 4 | 5,105 | 6 | – | – | – | *mbeA, mbeC* | – | – | – |
| V08-21-A02-002-010 | Healthy pig | pA287 | AMP CTX CHL COL STR NAL TET SXT | O | AMP CTX TET SXT COL | – | Chromosome | 5,017,435 | 4,603 | – | – | *arsA,arsB,arsC,arsD,copA,corA,cueO,cueR,cusA,cusB,cusC,cusF,cusR,cusS,rcnA,rcnB,rcnR,zitB,zntA,zntR,zupT* | *IS1397,IS1A,IS1F,IS1R,IS200C,IS3411,IS4,IS609,IS629,IS911,ISCep1,ISEc1,ISEc17,ISEc31,ISEc38,ISEc46,ISEc8,ISLad1,ISRor2,ISSd1,ISSen7*,*ISSfl10,ISYps3,intA,intQ,intS* | *chuR,chuW,ecpA,ecpB,ecpC,ecpD,ecpE,entA,entB,entC,entD,entE,entF,entH,entS,fepA,fepB,fepC,fepD,fepE,fepG,fes,fimA,fimC,fimD,fimF,fimG,fimH,flu,fyuA,hcp,hcpA,hemR,hemS,hlyE,hmuT,hmuU,hmuV,invA,invF,lpfA,lpfD,mxiC,ompA,ompC,ompD,ompF,ompX,papC,papD,prgH,prgI,prgK,sctC,sfmA,sfmC,sfmD,sfmF,sfmH,spaL,spaO,spaP,spaQ,spaR,spaS,vgrG1* | *dnaA,dnaB,dnaC,dnaE,dnaG,dnaN,dnaQ,dnaX,ftsA,ftsI,ftsK,ftsL,ftsN,ftsQ,ftsW,ftsZ,gyrA,gyrB,ligA,ligB,mukB,mukE,mukF,parC,parE,polA,polB,rep,seqA,topA,zipA* | *cbeA,cbtA,ghoS,ghoT,hicA,hicB,higA,higB,hipA,hipB,hokE,prlF,yhaV* |
|  |  |  |  |  |  | IncHI2A, IncN, IncHI2 | Plasmid 1 | 255,298 | 281 | – | *sul2, mcr-3.1, bleO, aph(3'')-Ib, aph(6)-Id, aph(3')-Ia* | *silE, cusS, cusR, cusC, cusF, cusB, cusA, silP, copA, copB, pcoC, pcoE* | *ISEcl1, traC, ISKpn40, IS903, IS103, IS150, ISVsa3, IS1006, TnAs3, ISEc63, tnsA, tnsB, TnAs1, IS26, ISAba1, ISAba33, TnAs2, IS1R* | *virB, hha, hns, yceD, alx, stiP* | *repB, parM, uvrD, pvuIIM, dsbC, umuC, umuD, mepM, smc, dcm, repE, dgkA, dam* | *hipA, higB-1* |
|  |  |  |  |  |  | IncY | Plasmid 2 | 84,415 | 84 | – | qnrS1, *bla*_CTX-M-15_, *bla*_TEM-1_, *aph(6)-Id, aph(3'')-Ib, sul2, dfrA14, tet(A)* | – | *IS26, IS1X3, IS1X4, IS4321R, ISRaq1, TnAs1, IS26, xerC, hin, ISKpn19, hin, ISEc36, Tn2, ISEcp1, Tn2, tnpR, IS5075, ISPa38, IS26, xerC, IS26, TnAs1, TnAs1, xerC, IS1R, IS1A, traC, ISSbo1* | *virB, lon, VirB11, clsB* | *repA, parA, topB* | *relE2, relB, relE* |
| V05-23-A02-002-010 | Healthy pig | pA2111 | AMP CTX CIP COL | O | AMP CTX COL | – | Chromosome | 4,733,001 | 4,369 | – | – | *zntA,feoC,feoB,feoA,zntR,mntH,rcnB,rcnA,rcnR,znuB,znuC,znuA,mntR,mntS,zitB,cusA,cusB,cusF,cusC,cusR,cusS,cueR,copA,cueO,corA* | *IS1A,IS1H,IS1X2,IS3,IS3411,IS609,IS621,IS629,ISCep1,ISCfr6,ISEc1,ISEc17,ISEc26,ISEc38,ISEc45,ISEc5,ISEc52,ISEc78,ISRor2,ISSen7,ISVsa17,ISVsa5,ISYps3,intA,intS* | *bcsG,bcsE,bcsQ,bcsA,bcsB,bcsZ,bcsC,papD,papC,spaO,spaP,spaQ,spaR,spaS,prgH,orgA,ompC,fimA,focC,fimD,fimF,fimG,fimH,chuR,ompD,ompN,ompG,ompX,hlyE,csgC,csgA,csgB,csgD,csgE,csgF,csgG,pgaA,pgaB,pgaC,pgaD,ompA,ompF,entH,entA,entB,entE,entC,fepB,entS,fepD,fepG,fepC,fepE,entF,fes,fepA,entD,ecpR,ecpA,ecpB,ecpC,ecpD,ecpE,fimC,lpfA,lamB* | *dnaA,dnaN,recF,gyrB,recG,topA,dnaG,parE,parC,mutH,recC,recB,recD,mutS,recA,recN,recO,ftsA,zipA,gyrA,uvrC,mukB,mukE,mukF,ftsK,uvrB,dnaE,ftsZ,ftsQ,ftsW,ftsI,ftsL,dnaC,mutL,ssb,uvrA,dnaB,ftsN,uvrD* | *cbeA,cbtA,dinJ,ghoS,ghoT,hicA,hicB,higA,higB-2,hipA,hipB,hokE,mazE,mazF,prlF,vapB,vapC,yafQ,yefM,yhaV,yoeB* |
|  |  |  |  |  |  | IncFIB,FIC | Plasmid 1 | 156,022 | 169 | – | *aadA1,* multiple *bla*_TEM_ variants (6), *cmlA1, qnrS1, sul3, tet(A), dfrA15* | *yciC,mntB* | *xerC,TnAs3,hin,tnpR,Tn2,TnAs1,pinR,traI,traD,traQ,traN,traC,traR,traV,traA,traY,traJ,traM,ssb,is629,is1r,is3411,is2,iskpn28,is26,is406,isssu9,iskpn19,isec36* | *ompT,fes,pfeA,cvaC,cma,cmi,rdmC,aidA*,*iutA,iucD,iucC,iucB,iucA* | *repB,cai,hcaB,finO,ylpA,psiB,sopB,crcB,eno,umuC* | *cia,pemK,pemI,vapB,vapC* |
|  |  |  |  |  |  | IncB/O/K/Z | Plasmid 2 | 97,524 | 103 | – | *aph(3’’)-Ib, aph(6)-Id,* *bla*_TEM-1C_, *FloR, sul2* | – | *ssb,ISRor2,Tn2,tnpR,ISVsa5* | *hdfR, bfpB, rfaH* | *repA,umuC,umuD,yhdJ,klcA,noc,psiB* | *pndA* |
|  |  |  |  |  |  | IncI2 | Plasmid 3 | 72,329 | 87 | – | *mcr-1.1* | *zinC* | *IS103,ISSbo1,IS150,traL,topB,xerC,ISSfl3,traG,rcbA,ISPsy42,hin,yheS,mobC,nikB* | *proQ,ymoA,sctC,VirB11,virB8,virB4,virB1,relE* | *repA,flmC,ybbJ,qmcA,pap2* | – |
|  |  |  |  |  |  | IncFII | Plasmid 4 | 64,681 | 76 | – | *bla*_CTX-M-27_ | – | *IS1D, IS26, IS15, traM, traY, traA, traV, traC, traN, traQ, traD, traI* | *aidA, yhcR, hha* | *stbB, parM, yhdJ, klcA, ssb, noc, psiB, flmC, ylpA, finO* | *pemI, pemK* |
|  |  |  |  |  |  | rep_cluster_2350 | Plasmid 5 | 6,647 | 8 | – | – | – | *mbeA, mbeC* | – | *rop* | *cea* |
|  |  |  |  |  |  | Col440II | Plasmid 6 | 5,429 | 4 | – | – | – | *mbeA, mbeC* | – | – | – |

AMP, ampicillin; CHL, chloramphenicol; CIP, ciprofloxacin; COL, colistin; CTX, cefotaxime; NAL, nalidixic acid; STR, streptomycin; SXT, trimethoprim/sulfamethoxazole; TET, tetracycline. CDS, coding DNA sequences.
